# Deep2Full: Evaluating strategies for selecting the minimal mutational experiments for optimal computational predictions of deep mutational scan outcomes

**DOI:** 10.1371/journal.pone.0227621

**Published:** 2020-01-10

**Authors:** C. K. Sruthi, Meher Prakash

**Affiliations:** Theoretical Sciences Unit, Jawaharlal Nehru Centre for Advanced Scientific Research, Bangalore, India; UMR-S1134, INSERM, Université Paris Diderot, INTS, FRANCE

## Abstract

Performing a complete deep mutational scan with all single point mutations may not be practical, and may not even be required, especially if predictive computational models can be developed. Computational models are however naive to cellular response in the myriads of assay-conditions. In a realistic paradigm of assay context-aware predictive hybrid models that combine minimal experimental data from deep mutational scans with structure, sequence information and computational models, we define and evaluate different strategies for choosing this minimal set. We evaluated the trivial strategy of a systematic reduction in the number of mutational studies from 85% to 15%, along with several others about the choice of the types of mutations such as random versus site-directed with the same 15% data completeness. Interestingly, the predictive capabilities by training on a random set of mutations and using a systematic substitution of all amino acids to alanine, asparagine and histidine (ANH) were comparable. Another strategy we explored, augmenting the training data with measurements of the same mutants at multiple assay conditions, did not improve the prediction quality. For the six proteins we analyzed, the bin-wise error in prediction is optimal when 50-100 mutations per bin are used in training the computational model, suggesting that good prediction quality may be achieved with a library of 500-1000 mutations.

## Introduction

Mutations are changes in the nucleotide sequence of an organism, and its effects may be noticeable across the scales from protein expression, cellular or organismal level. Most mutations are usually found to be neutral or deleterious across the scales, while a very few of them turn out to be beneficial i.e. confer an increase in phenotypic fitness. [1] Interestingly a very large portion of genetic variation in eukaryotes is represented by single nucleotide polymorphisms (SNPs), [2, 3] which is at most a variation in a single amino acid in a protein. Various disorders, such as diabetes or cancers, [4, 5] and public health concerns such as antibiotic resistance can be traced back to such single mutations [6–8] in key human or bacterial proteins respectively. Thus, it is important to be able to predict the phenotypic effects of new mutations. However, exploring the mutational landscape is resource intensive, involving introduction of mutations, expression and purification of the proteins and characterization of their functional effects *in vitro*. Despite this difficulty, hundreds of single point mutations, site-directed or random mutations were performed on many interesting proteins. Alanine scan mutagenesis [9] emerged as a systematic and popular biochemical technique. In alanine scan, all potentially interesting amino acids are replaced with alanine, which is a small and neutral amino acid and studied for the effects on stability and function of the protein. The beneficial or detrimental effects of mutations on binding, [10] stability, [11] enzymatic activity, [12] etc., were used to dissect sequence-function relationships.

While mutations at the active sites of enzymes are relatively easy to interpret, understanding how distal mutations affect the catalytic activity is a challenge on its own. Predicting a change in cellular or organismal fitness upon a single mutation in proteins is further complicated, since fitness is a downstream effect and an immediate correlation with changes in structural stability and dynamics of the protein may not be easy. However, such an understanding will have an enormous impact, whether it is for identifying disease causing mutations in the human genome or for designing antibiotics. The development of high-throughput technologies has driven newer and massively parallel approaches in the exploration of mutational landscapes at a cellular phenotypic level. Methods such as deep mutational scan [13, 14] or site saturation mutagenesis [15] now made it possible to study the fitness consequences of a very large number (∼ 10^5^) of independent mutations of the same protein. The mutational effects from these extensive mutational scans are important from the perspective of basic biology to understand how proteins work as well as for protein and drug design.

Deep mutational scanning experiments explore the cellular phenotypic effects of thousands of mutants by way of massive sequencing. [16] In principle, the methodology involves an extensive and exhaustive single point mutational scan, generating libraries of variants where every single amino acid in the protein is replaced with all 19 alternative possibilities. To be consistent with the terminology used in this field, we refer to these possible variants as mutations. While several deep mutational scan studies have demonstrated the utility of the method in analyzing the effects of large number of mutations, performing such experiments is highly resource demanding. Further, as the interest in the study of simultaneous multiple mutations increases, such as in the case of drug resistance and compensatory mutations, [17] the number of mutational studies required will increase by orders of magnitude. Thus, alternative or complementary approaches that quantify the fitness effects of a wide range of amino acid mutations have to be developed.

Several computational tools have been developed to predict the functional effects of mutations: SIFT [18] is based on evolutionary information obtained from the sequences of proteins and their homologs whereas SNAP2 [19], PON-P2 [20] use other features such as functional annotations along with evolutionary information. Tools such as SNPs3d [21] and Polyphen [22] use information about the 3D structure of the protein also. Condel [23], CADD [24], REVEL [25] and PON-P [26] are predictors that combine the predictions of other tools. Unsupervised methods using sequence covariation (EVmutation) [27] proposed statistical energy scores to be correlated to the fitness effects of mutations, and newer developments in this methodology (DeepSequence) [28] exploit the latent variables to improve the predictions. Recently, deep mutational scan data from different proteins was used for developing a global quantitative model for mutational effects predictions (Envision), [29] which was then used for predicting the effects of all possible single amino acid substitutions in the proteomes of human, mouse, frog, zebrafish, fruit fly, worm, and yeast.

All these models except EVmutation, [27] DeepSequence [28] and Envision [29] act as classifiers, and none have the flexibility to adapt when the assay conditions are changed. There have been Proteins Specific Predictors (PSP) [30] which are developed by training on data of specific proteins to classify mutations. Making quantitative predictions of the downstream effects of mutation under an external selection pressure is not easy. While it may be too soon for computational methods to completely replace wet-lab experiments, they are certainly at a stage where they can be used to reduce the number of experiments required and hence the costs of generating such large data sets. The next paradigm in the evolution of the models is thus a combination of partial data from deep mutational scans with computational models. Recently it was demonstrated that the large fractions of data missing from mutational scans can be imputed [31, 32] using machine learning approaches. It is thus clear that exploiting the information about the system and the mutations, one can predict the effects of missing mutations. Continuing on a similar theme, we explore a complementary question about better ways of designing DEEP mutational scan to develop predictions for a FULL mutational scan (DEEP2FULL). Specifically we ask if there is a better strategy to design the experiments with minimal number of mutations and to prioritize experiments rationally, and yet achieve the best possible predictions for the rest of the mutations. In this work, we use publicly available deep mutational scan data on six proteins and illustrate the outcomes of a few strategies we define for choosing the minimal set of mutations.

## Results

### Neural network models for predicting fitness

To computationally predict the outcomes of deep mutational scans we developed artificial neural network (ANN) models, using variables which can describe the physico-chemical properties of the wild type amino acid and the substitutions, and partial experimental data on the fitness consequences of the mutations. Seventeen different descriptive parameters (Methods section), including 4 parameters derived from the protein structural information, 7 variables from sequence information and 6 others from co-evolutionary information were used in our models. The experimental data we used consisted of relative fitness of the mutant cells with respect to the wild type under selection pressure from different stressors or their concentrations. ANN is similar in philosophy to the goal of predicting the downstream effects of the mutations, as the clarity of what happens at the intermediate stages, also known as layers, is compromised in favor of the end results it generates. While it lacks the simplicity of a linear regression model, it can in principle embody all the complex non-linear interactions that occur at the different stages of the effect propagation, starting from the mutation and ending with the change in fitness. Although several machine learning approaches such as random forests [31] may be useful for making predictions, we chose to work with artificial neural networks. We used feedforward neural network with Levenberg-Marquardt back-propagation algorithm implemented in the Neural Network Toolbox of Matlab along with the early stopping criterion for termination of training. For each data set chosen for modeling, neural network models were built by subdividing it into training, validation and test sets. Apart from the input and output layers, all neural networks had a single hidden layer and the number of neurons in this layer was chosen based on the coefficient of determination (*R*^2^) for the training and validation set predictions (Methods).

### Impact of sampling size on the model’s predictive ability

**Fig 1:**
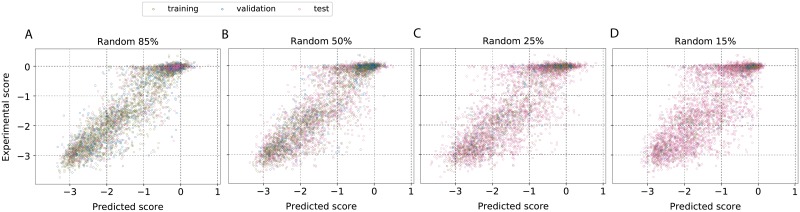
Systematic increase of training data size improves prediction quality. The experimental data on the relative fitness of *E. coli* with mutations in β-lactamase was modeled. The fraction of the complete data that was used for training and validation was systematically reduced in four steps from 85% to 15% to see how the quality of computational predictions of fitness changes. It can be seen that the quality of predictions when trained with 50% is comparable with the one trained at 85% data. The prediction quality is tabulated in Table 1 in S1 File. Results from predictions of other proteins are in Figs 3-5 in S2 File.

**Fig 2:**
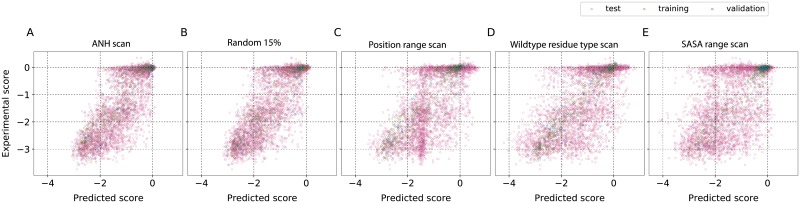
Representation of the types of mutations in the training set influences the results. A comparison of the different strategies we used for choosing the training set with 15% data completeness. In an extension of the concept of alanine-scan, the fitness outcomes from alanine (A), asparagine (N) and histidine (H)-scans at each amino acid position were used as the training set, and the fitness scores for all other 17 mutations at every site were predicted. The results were compared to other strategies that used random (Random 15%) or site-directed protocols (position range scan, wild type residue type scan and SASA range scan) for choosing the minimal set required for training. The results suggest that choosing mutations randomly or performing an ANH scan is better than scanning all mutations at a few positions.

**Fig 3:**
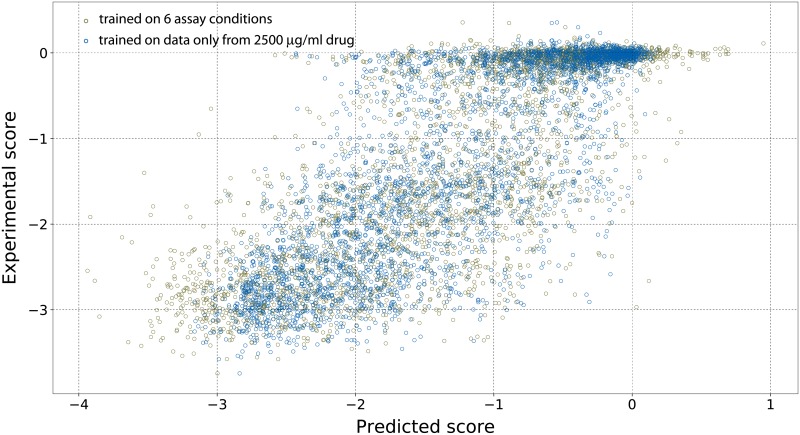
Augmenting with scores at different assay conditions did not improve predictions. At 15% mutational completeness, the data size was augmented by combining data from six different assay conditions. There was no improvement in the prediction quality although the data was enhanced 6-fold. The $R_{test}^{2}$ with and without data augmentation was 0.61 and 0.66 respectively. More detailed results are in Fig 10 in S2 File. Similar analysis was performed by developing model trained on data from the 650 μg/ml drug concentration assay. In this case also the predictive ability of the models trained at only one concentration or at multiple concentrations was similar.

**Fig 4:**
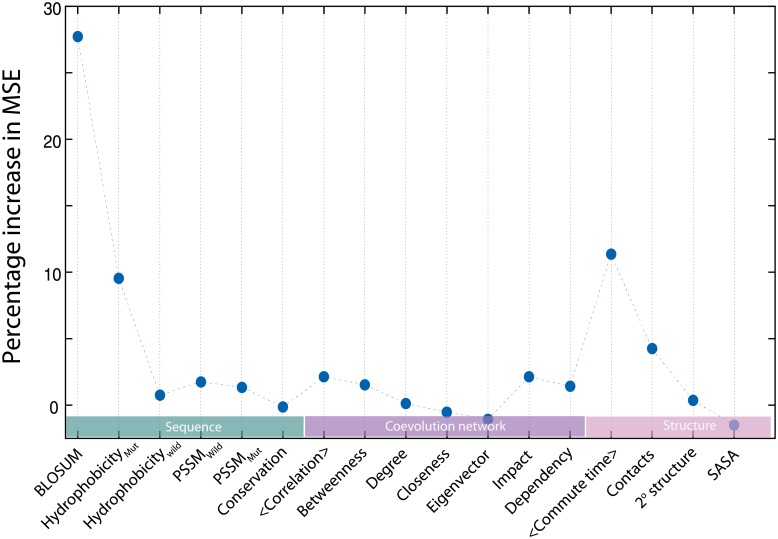
A few variables contribute significantly. The relative importance of different variables in the predictive model trained with 85% data from β-lactamase mutations was evaluated. The sensitivity of the model to a variable was quantified as the percentage increase in the mean squared error (MSE) between the prediction and the experimental values when the variable was replaced with its average calculated across all mutations. BLOSUM score, average commute time and hydrophobicity of the mutant have the highest contribution while some of the variables have little contributions in the model. None of the variables we used is perfectly correlated to any other variable, however, the poor contributions suggest that they could be correlated to a non-linear combination of other variables.

**Fig 5:**
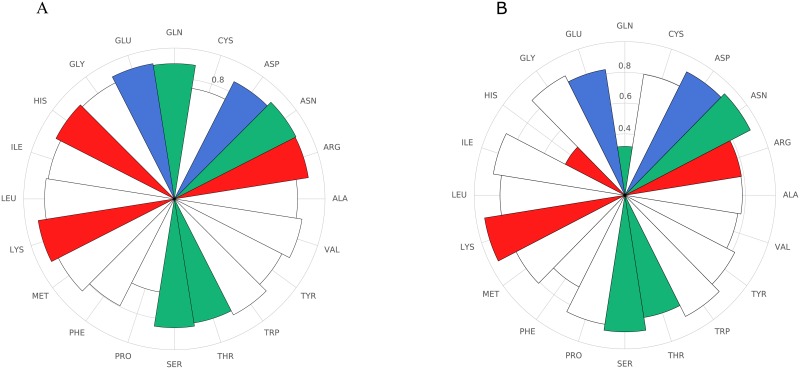
Random scan obtains comparable predictions for different amino acids. The test set of random 25% scan was sorted based on the amino acid after mutation and the amino acid in the wild type. The quality of predictions as quantified by Pearson correlation is shown for (A) the amino acid after mutation (B) the amino acid in the wild type. Amino acids are colored according to their type: red (positively charged), blue (negatively charged), green (polar), white (hydrophobic). The random scan results in roughly uniform quality of predictions for all substituted amino acids.

The first strategy we evaluated was a systematic reduction of the size of the experimental data that was used to train the model. From the complete mutational scan data that was available, a set of randomly chosen variants was used for training and validation and a systematic reduction in the size of this set (85%, 50%, 25% and 15%, respectively) was made for developing four different models. We analyzed six proteins—β-lactamase, [33] aminoglycoside 3’-phosphotransferase (APH(3′)-II), [34] heat shock protein 90 (Hsp90), [35] mitogen-activated protein kinase 1 (MAPK1), [36] ubiquitin-conjugating enzyme E2 I (UBE2I) [31] and thiamin pyrophosphokinase (TPK1). [31] The selection was based on the criterion that data on at least 2500 mutations are available although the assays measured different phenotypes. Some of these experiments measured a change in the average rate of cell division upon mutation, [31] while others measured a consequent variation in the population. [33] The predictions of the model developed using 85% of data for β-lactamase is shown in Fig 1 in S2 File and a comparison of the same with experimental data in Fig 2 in S2 File. Results from models trained on smaller data sets are summarized in Fig 1 for β-lactamase and for the other five proteins in Figs 3-5 in S2 File. As expected, the overall quality of predictions improves with increase in the data used for training although the improvement is sublinear (Table 1 in S1 File). As can be seen from these results, except for the case of TPK1 the Pearson correlation between predicted and experimental fitness begins to saturate when more than 50% of the data is used for training the model.

### Comparing complementary ANH and four other mutational scans trained on 15% of data

One common feature of the four models developed above is that they are all trained on mutations randomly selected from across the sites and possible substitutions (random scans). We further explored if using systematically chosen mutations in model development can help improve the prediction quality. We performed these analyses with the smallest amount of data to have a better chance of observing the differences. We first used the fitness scores from alanine scan, and predicted the outcomes for all other 19 mutational scans. However, in our search for the minimal and predictive data set, alanine scan data was not satisfactory ($R_{test}^{2}=0.38$, Fig 6 in S2 File). Hence we performed other comparative analyses starting with an augmentation of the alanine scan. It was recently discovered [37] that in multiple deep mutational scan data sets the fitness changes upon mutation to any amino acid is best correlated statistically with the fitness scores associated with asparagine (N) and histidine (H) substitutions. Taking a cue from this observation, we combined the commonly used alanine (A) scan, with asparagine (N) and histidine (H) scans, thus choosing one from each charge type—hydrophobic (A), polar (N) and charged (H), to develop an ANH scan. We then used ANH mutational scan data which is 3/20 or 15% of the full mutational scan data, as a strategy to train the neural network model and to predict the remaining 17 amino acid scan results at every site. The ANH scan data was further divided as 85% for training and 15% for validation of the model. As seen in Fig 2A and Table 2 in S1 File, the fitness predictions improve relative to the one obtained by training on alanine scan data ($R_{test}^{2}=0.62$). The results from training the models with either ANH-scan or a random scan, both with 15% data, are comparable, with one working slightly better than the other depending on the protein.

We explored a few other systematic mutagenesis schemes based on the concept of site-directed mutagenesis. We asked if having the data for all 19 mutations at a few positions could improve the prediction quality. We used three different ways of identifying these positions: 1) *Position range scan*—Residue positions were randomly chosen to have an approximately uniform sampling of the sites along the primary sequence; 2) *Wild type residue type scan*—Depending upon the distribution of wild type amino acids, in this scan wild type positions were chosen to ensure that there is a nearly uniform representation of the 20 amino acids in the training set; 3) *SASA range scan*—Residue positions were chosen in such a way that the distribution of solvent accessibility is uniform over the training and validation sets. The idea was to have representation from the residues with different levels of solvent exposure in the training set. The results for β-lactamase are shown in Fig 2 and those for the other five proteins are summarized in Figs 7–9 in S2 File. The results for all three position based scans, all trained on 15% data, were poorer than those from a random or ANH scan.

### Augmenting data with transverse assay conditions

We also investigated whether with the same number of mutations, the prediction quality could be improved by using transverse data from different assay conditions. The rationale for evaluating this strategy was to compensate for the number of mutants with the number of cultures with different stressor concentrations. We trained our models using 15% of the mutational data, but with the fitness changes measured at six different drug concentrations, [33] thus enhancing the total data used for training by 6-fold. Plotting the fitness change for each mutation with *log [ampicillin]* displayed a regular sigmoidal pattern in the dose-response curve, thus raising the possibility that the augmentation brings more structured data and improves predictability. We compared the mutational effects predictions for the studies at 2500 μg/ml using two models, one trained on data from six different concentrations and the other trained only on the data from experiments performed at concentration 2500 μg/ml. However, contrary to our expectations as shown in Fig 3, there was no significant difference in the prediction quality by using data at different concentrations for training. The same was also true for the predictions of the mutational effects at 650 μg/ml drug concentration.

### Variable importance and models with fewer variables

The primary aim of the work was to reduce the experimental data needed for building the model. However, conceptually it is also interesting to ask if the role of different predictive variables used in the model can be quantified and if the model itself can be simplified. We illustrate the relative importance of the different descriptive variables using our calculations on the fitness (dis)advantage in *E. coli* exposed to ampicillin, conferred by the single point mutations in TEM-1 β-lactamase, [33] although the scope of the analysis is general. For investigating the contribution of individual input variables in the predictions, the input variable was kept fixed at its mean value for all the samples and the network was retrained. The change in mean squared error (MSE) on the removal of a variable is used for quantifying the importance of that input variable. Fig 4 shows the difference in MSE when each variable is replaced by its mean value. BLOSUM which represents the substitution effects based on evolutionary data has the highest contribution to the predictions. Hydrophobicity index of the amino acid to which the mutation is made and the average commute time are the other variables with significantly higher contributions. In addition to the 17 variables, we also added the statistical coupling energy [27] as an additional variable to see if it improved the correlation between the predictions and the observations. No improvement was noticed, possibly because other variables including the ones from co-evolution data already implicitly accounted for this factor (Table 3 in S1 File). Since the proximity of an amino acid to the catalytic site could be of high functional significance, we developed a model with this factor as an additional descriptive variable. The catalytic residues were identified in β-lactamase and the distance of every amino acid to the nearest catalytic residue was computed. This additional input variable did not improve the predictions either. As in the case of statistical coupling energy, the information contained in this variable could be represented by other variables like conservation, number of contacts and commute time. So statistical coupling energy and distance from catalytic sites were not used in any other analysis in this work. We also analyzed the contributions at a coarse level, creating neural network model for alanine scan mutations using only (1) sequence based variables and (2) structure based variables. The sequence based model performed better than the structure based one, *R*^2^ values being 0.54 and 0.25 respectively for the sequence and structure based models for the test set chosen from the alanine scan data set.

We selected fewer variables and developed minimal models using two different measures to rank the individual variables: Pearson correlation of the individual variables with the measured fitness and the change in MSE on replacing the variables with their averages. Using these two criteria models were developed using 7 and 6 variables respectively (Methods section). Average correlation, average commute time, number of contacts of the wild type amino acid, and BLOSUM score for the substitution were the most relevant variables according to both of these criteria. The results obtained (Fig 11 in S2 File) from these two reduced models are of comparable quality to the ones constructed with 17 variables. However, in the interest of the scope of the present work which is about reducing data rather than reducing variables all our analyses are presented with the results of model trained on 17 predictive parameters.

### Quality analysis of output and input

The quality of predictions in our analysis was verified based on three different measures—(1) the overall $R_{prediction}^{2}$, Root Mean Square Deviation (RMSD) and Pearson correlation, all three metrics suggested that the quality of our predictions were comparable with other models which use partial data for prediction (Table 4 in S1 File). It is notable that *R*^2^, which is very sensitive to outliers also had shown that the predictions are reasonable even at low data completeness, (2) the prediction data was segregated either based on the amino acid before or after mutation. The outcomes for some of the amino acids are relatively poor as seen from the individual regression plots (Figs 12 and 13 in S2 File). Amino acid wise prediction quality can be summarized using their Pearson correlation values also as shown in Fig 5. We further analyzed and found that the quality of predictions for different amino acids (Fig 5B) was not correlated with their frequency in the training set. It can be seen that the effects of some amino acid mutations do not span the entire range of fitness scores, hence predictions could not be improved. (3) For a predicted fitness, the variation in the experimental values. This is summarized in Fig 2 in S2 File with histograms of experimental fitness generated from the predicted fitness variation around -3, -2, -1 and 0. While these histograms show a variation relative to the predicted fitness, it must be noted that even in different trials of the experiment, there is a significant variation. We also investigated the prediction quality for amino acids with different solvent exposure (Fig 14 in S2 File). As can be seen, in general the predictions were better in quality for the solvent exposed residues. This could be because of the lower variability in fitness scores at higher SASA range.

Across the six proteins we studied, the quality of the predictions varied. We checked if it is possible to define a measure for the quality of input data which forces a requirement on the size of the data set used for training. It is apparent from the experimental data that the range of measured fitness varies depending on the protein and stressor concentration. It also appears from our results that the prediction quality may be slightly better when the fitness effects in the experiments span a broader range. In an attempt to clarify these effects of input data quality and size, we defined a quality metric of the input data as the ratio of the range over which the training data spans and the standard deviation of the data centered around what appears to be the neutral mutations. The motivation for choosing such a metric is that the wider range of mutational scores and the separability of neutral mutations from the rest will lead to improved predictions. Mutational effect scores for β-lactamase measured under different concentrations of ampicillin (2500 μg/ml, 625 μg/ml, 156 μg/ml, 39 μg/ml) were available in the study by Stiffler *et al*. [33] We developed models for each of these data sets and the quality of inputs and output is plotted in Fig 15A in S2 File showing a correlation that, as the quality of the input data increases, the prediction quality improves as well. Similar input-output quality analysis was made for all proteins and random scans with systematically increasing data as shown (Fig 15B in S2 File). The results although not very conclusive suggest that the predictability of the fitness may be improved by using data obtained at high stressor concentrations.

## Discussion

### Scanning strategies: Training with reduced data

The goal of Deep2Full was to evaluate how the data required for developing the computational model can be systematically reduced, and if for a given size of data a systematic sampling can improve the quality of predictions. Having additional data of fitness scores from other assay conditions such as different drug concentrations with the smaller training sets did not improve the prediction quality. Quite intuitively, the quality of prediction for all the proteins improves with increase in the training data from 15% to 85%. We used two different metrics of quality—RMSD and Pearson correlation. Although the prediction quality increases, the improvement does not scale linearly with the data size (Fig 16 in S2 File). With both these measures we could see a sign of saturation when more than 50% of the data was used for training. We also analyzed the error across the range of experimentally measured fitness. Ten bins of equal widths were created dividing the experimentally observed fitness in each protein and RMSD for the test set was calculated for each bin. For all the proteins, the error systematically increases as the expected effects of mutations increase (Fig 17 in S2 File). To further understand this RMSD, we plotted RMSD for each bin relative to the number of data points in this range that were used for training the model (Fig 18 in S2 File). The two observations that come out are a power-law behavior in the error and that increasing training data from each bin beyond 50 to 100 did not improve the predictions significantly. This optimal choice along with a 10-bin division suggests about 500-1000 mutations to be required for developing neural network models. However it appears that one can increase the data used for training in each bin selectively to achieve this optimality. This training data size is approximately 15-25% of the data in the cases we studied.

### Scanning strategies: Choosing mutations for training

We asked if there is a better way of choosing the mutations that are used for training the model, guided either by the physico-chemical factors or on the experimental ease of obtaining those mutations. We performed these analyses at the lowest level of data (15%) that yields reasonable predictions, the rationale behind it being that any differences in the strategies will be pronounced and easy to infer. A consistent pattern noted in our study of all six proteins is that at 15% completeness, randomly selecting variants as well as the systematic ANH scan yielded results of comparable quality. The alternative of training the model by selecting all 19 substitutions at a few positions, selected for a representation across SASA or wild type amino acid range were poorer in predictive ability. These trends were consistent in the different metrics we used for determining the quality of predictions—RMSD and Pearson correlation (Table 2 in S1 File). It is clear from this analysis that in developing a model, a training set having representation from every position in the protein is much more valuable than having all substitutions at a few sites.

The underlying objective of this exercise was to see if the efforts of constructing the mutant libraries, clones and sequencing can be reduced, without compromising on the quality of the learning. In our model, a random scan implies a random and unbiased choice of the mutation from across the primary sequence where a transition from any amino acid to another is feasible, such as the ones that could be achieved with mutagenesis techniques like POPCode [31] and single-site saturation mutagenesis. [38] We also investigated another scenario where the SNPs may be generated by an error-prone PCR (epPCR), which has an inherent bias against certain mutations. [39, 40] When the model was trained on data set same in size to that of Random 25% scan, but the mutants being chosen from SNPs which were theoretically considered achievable by epPCR, [41] the prediction quality was comparable (Fig 19 in S2 File and Table 1 in S1 File) for all the six proteins we studied. A detailed cost-benefit analysis considering the number of experimentally realizable mutations which could range from 15% to 85% of data completeness and the accuracy of predictions, will be required to choose between epPCR and site-directed mutagenesis techniques for generating mutant libraries. [42]

### Need for hybrid models

Scoring models such as SNAP have been used for classifying the mutations as fitness-neutral or non-neutral. [44] Other recent co-evolution based models have shown a good correlation of the fitness variations observed in deep mutational scans with the predictions of the evolutionary statistical energy [27] and DeepSequence [28]. As shown in Fig 20 in S2 File, for the specific example considered, the relations between the scores and cellular fitness using 15% and 50% of randomly selected mutations are at least as noisy or worse than the models we used. Envision [29] was a model that was ambitiously developed to make unsupervised predictions for the deep mutational scan. The model was validated using leaving-one-protein-out protocol. However, as it can be seen from Table 5 in S1 File, when using the model for a newer protein such as TPK1 and UBE2I the predictions were not satisfactory. This limitation of the generalized model may be more likely because the mutational effects in proteins are complicated to predict, rather than because of the shortcomings in the specific model. As noted in Table 5 in S1 File some of the unsupervised computational predictions that are reported in the literature had good correlations with the experimental data. This raises a question on why the present work focuses on hybrid computational models with minimal experimental data, when the models may possibly be developed with no experimental data. From the deep mutational scan studies on β- lactamase, [33] one can see that the fitness outcomes change with the stressor concentration and with the type of the stressor (stressors: cefotaxime and ampicillin). It was also highlighted in the mutational studies of APH(3′)-II [34] that the fitness landscape depends sensitively on the type of the antibiotic, even when all of them are believed to interact with the same active site. A model that does not use partial experimental data will certainly be insensitive to these differences in the assay conditions. Further, compared to a generic model, one may be able to use highly descriptive protein-specific variables which may improve the predictions.

Newer hybrid models [31] combined partial experimental observations along with other biophysical descriptors to impute the missing data. They could establish that it is possible to achieve predictions that are comparable to the experimental variance across the trials. We used a similar approach of hybrid model and obtained predictions for TPK1 and UBE2I comparable with those from other works [31] (Table 4 in S1 File). The predictive ability of our models for the proteins β-lactamase, APH(3′)-II and Hsp90 are also comparable with that of Envision, where 80% of data was used to develop models for individual proteins [29] (Table 5 in S1 File). The hybrid models and the strategies will gain prominence as the experimental emphasis shifts towards simultaneous multiple mutations. However, in the present hybrid approach using experimental data and computations one encounters at least two disadvantages that expertise in building computational models and experimental data are required for model development.

### Scope and limits of Deep2Full

Although the results of our computational predictions are comparable to those from other hybrid models, the focus of the present work was different. Our goal in this work was not to validate whether the missing data can be predicted, but rather to evaluate if there is a rational way of planning the reduced experimentation. The scope of Deep2Full was to conceive a few ways of designing the minimal number of experiments that will be helpful in training models and evaluating their efficacies in making the predictions for the complete set of variants. It appears that a randomized set of mutations presents the best training set, followed by a charge based training set. While it is preliminary to say, it appears that by optimizing the stressor concentrations in the assays, one may be able to obtain comparable quality of results with fewer mutations. A re-evaluation of the strategies considering the costs or convenience associated with them may be a subject of future work.

## Conclusions

Deep2Full was developed in the context of a new paradigm of hybrid models that train the computational models with partial deep mutational scan data from across assay conditions, to quantitatively predict the fitness outcomes of a full-set of mutations. By combining this phenotypic deep scan data with structure, sequence and co-evolutionary information, the possible outcomes of a full set of deep mutational scan were predicted. We addressed two questions, how much the data size used for training can be reduced and if there is a better way of performing these mutations. To train the models, we found that a representation from all positions of the protein was required. The neural network models which were constructed with seventeen variables from—structure, sequence or co-evolution could in principle be simplified by as few as seven variables, although the model reduction was not the emphasis of the present work. Variation in the experimental data in the different trials, and the choice of the phenotype being measured, such as the differential rate of growth or changes population size, are the limitations the model begins with and this quality of data certain imposes constraints on the size of the data that is necessary for training a reliable model. Regardless, it appears that the best way to enhance the prediction is with a random scan of the sites and substitutions.

## Methods

### Data sets chosen

Deep2Full was developed for the deep mutational scan data of 6 proteins: β-lactamase [33], aminoglycoside 3’-phosphotransferase (APH(3′)-II) [34], heat shock protein 90 (Hsp90) [35], mitogen-activated protein kinase 1 (MAPK1) [36], ubiquitin-conjugating enzyme E2 I (UBE2I) [31] and thiamin pyrophosphokinase (TPK1) [31]. Data used for calculations involving mutational effect scores of β-lactamase at different concentrations of ampicillin was obtained from the study of Stiffler *et al*. [33]. Unless mentioned otherwise, the analyses on β-lactamase were preformed on the average of the two trials of the experiments at 2500 μg/ml concentration of ampicillin. For APH(3′)-II, Hsp90 and MAPK1, the computational models were built using the curated data from *Gray et al*. [37] Fitness scores for UBE2I and TPK1 were obtained from the study of *Roth et al*. [31] The total number of mutants used for developing model in each data set was: β-lactamase—3952, APH(3′)-II—4234, Hsp90—4021, MAPK1—4470, UBE2I—2563 and TPK1—3181. For the modeling efforts, we chose to work with proteins for which structural information and data on at least 2500 mutations were available.

### Division of data set

The variants for training and validation were chosen according to the strategy in each case. Fitness scores of the chosen variants were then grouped into 3 bins and data points in each bin were divided into training and validation sets in the ratio 85:15. For example, in the random 50% scan, we used 42.5%(= 0.85*50) for training and 7.5%(=0.15*50) for the validation set and the rest for testing. For the random scans the choice of mutations used for developing model is representative of the complete data set, as suggested by the similarity of SASA distributions for the complete data set and the training set which are similar (Fig 21 in S2 File).

### Choice of parameters

A total of 17 descriptive parameters were used in the our modeling. The *structural variables* for each amino acid that could be calculated from a reference protein structure were included in the model—(1) Solvent accessible surface area (SASA) (2) Secondary structural order, with a binary value 1 if the residue is part of a helix or β-sheet, and 0 otherwise (3) Number of structural contacts an amino acid has with a 4 Å cutoff (4) Average commute time, [43] which reflects the average connectivity of a given amino acid with the rest of the protein. The second group of independent parameters was based on the *sequence information*—(5) BLOSUM substitution matrix (BLOSUM62) score, which is the probability of substitution of an amino acid by other amino acids inferred from evolutionary information [45] (6) Hydrophobicity on the Kyte-Dolittle scale [46] of the amino acid after mutation (7) Hydrophobicity of the amino acid in the wild type (8) Position specific scoring matrix (PSSM) score for the amino acid after mutation calculated from the multiple sequence alignment (MSA) using PSI-BLAST (9) PSSM score for the wild type amino acid (10) Conservation of the amino acid. The third group of independent parameters was based on the properties of *co-evolutionary networks* that were constructed using the multiple sequence alignment (MSA) of hundreds of homologous proteins. This group is supposed to reflect the importance of an amino acid in an undirected co-evolutionary network—(11) Average co-evolution score of each amino acid (12) Degree centrality, the number of nodes to which a node is connected (13) Betweenness centrality, quantifying the importance of a node in connecting other pairs of residues (14) Closeness centrality, the inverse of the sum of distances to all other nodes (15) Eigenvector centrality, which considers not just the number of connections a node has, but also the connectivity of the immediately connected nodes. Directed network information is also included in the model—(16) Impact factor [47], the number of compensatory mutations required for mutations at a residue position is calculated based on conditional probabilities (17) Dependency factor, which is the counterpart of impact factor is the number of residues which are likely to influence a mutation at a given position. Details about calculation of these parameters are described in the following sub-sections.

### Multiple Sequence Alignment(MSA) and inputs calculated using MSA

For β-lactamase MSA was obtained from the Pfam database (Pfam ID: PF13354). Only 208 residues (positions 51–260) of the *E. coli* β-lactamase appeared in the Pfam alignment. So all the calculations and analyses were performed for the fitness effects of substitutions at these positions (3952 data points). For other proteins, homologous sequences were obtained through PSI-BLAST search and were aligned using Clustal Omega.

The variables calculated from MSA are:

*Conservation*: Conservation was calculated as the percentage occurrence of the most frequently occurring residue at a given position.
*Position specific scoring matrix (PSSM)*: PSSM was calculated from MSA using PSI-BLAST and it quantifies the probability of occurrence of each amino acid at each position of the protein.

### Co-evolution network and properties

Multiple Sequence Alignment (MSA) for the protein of interest was truncated to the reference sequence and sequences with a gap frequency less than 20% were used in the analysis. Consensus sequence was generated using the amino acid with the highest frequency at a given position. Following the Statistical Coupling Analysis protocol, [48] MSA was converted into a boolean sequence, with a 1 if the amino acid is the same as in the consensus sequence and 0 otherwise. *Undirected network*: The co-evolutionary relation between two amino acids *i* and *j* is calculated as proposed by Halabi *et al*. [48], *C*_*ij*_ = *ϕ*_*i*_ *ϕ*_*j*_|〈*x*_*i*_*x*_*j*_〉_*s*_ − 〈*x*_*i*_〉_*s*_〈*x*_*j*_〉_*s*_|, where $\phi_{i}=ln(({\langle x_{i}\rangle }_{s}\left(1-q^{a_{i}}\right))/(q^{a_{i}}\left(1-{\langle x_{i}^{s}\rangle }_{s}\right)))$, and $q^{a_{i}}$ is the probability with which the amino acid *a*_*i*_ at position *i* in the consensus sequence occurs among all proteins. *x*_*i*_ is the *i*^*th*^ column in the boolean sequence and 〈〉_*s*_ denotes the average over sequences. The co-evolutionary matrix is converted into a network representation using a cutoff *c*. If *C*_*ij*_ > *c*, we consider an undirected co-evolutionary network *i*—*j* to be present. In the present analysis weighted co-evolutionary matrix was used and the cut-off chosen was 1. We calculated different centrality measures—eigenvector centrality, degree etc. for the amino acid network described above, using the *igraph* module in python. [49]

### Directed network

Using the binary representation of the multiple sequence alignment, we created a directed influence network, in a co-evolutionary sense, with the following conditional probabilities:

$$\begin{array}{l} P(j=1|i=1)=\frac{\text{No}.\;\text{of}\;\text{sequences}\;\text{with}\;i=1\;\text{and}\;j=1}{\text{No}.\;\text{of}\;\text{sequences}\;\text{with}\;i=1\;\text{and}\;j=0\;\text{or}\;1}\\ P(j=0|i=0)=\frac{\text{No}.\;\text{of}\;\text{sequences}\;\text{with}\;i=0\;\text{and}\;j=0}{\text{No}.\;\text{of}\;\text{sequences}\;\text{with}\;i=0\;\text{and}\;j=0\;\text{or}\;1} \end{array}$$

where *i* and *j* represent positions. If both *P*(*j* = 1|*i* = 1) and *P*(*j* = 0|*i* = 0) are simultaneously greater than a value *P* (we used P = 0.8) then position *i* has an impact on *j*. A directed network is constructed by identifying all such pairs of residues. In this directed network, the number of outgoing links is considered the impact of an amino acid, and the number of incoming links is considered its dependency. The impact and dependency are supposed to summarize how many simultaneous mutations are forced or forced-upon by a mutation. [47]

### Average commute time

The hypothesis that the structural and dynamical connectivity of an amino acid to other amino acids determines the importance of an amino acid has been put forward. [43] The average commute time has been used for identifying hotspot amino acids. The resistance matrix is constructed using the number of atom-atom contacts between amino acids *i* and *j*, which are within 4 Å. The resistance matrix is then used for average commute time calculations as per the algorithm suggested in Ref. [43]. All structural variables including average commute time were calculated using the protein structure obtained using the Protein Data Bank (PDB) identifiers: β-lactamase—1M40, APH(3′)-II—1ND4, Hsp90—2CG9, MAPK1—4NIF, UBE2I—2UYZ and TPK1—3S4Y.

### Neural network model

All neural network calculations were performed using the Neural Network Toolbox of Matlab2017b. All neural network models had the architecture with an input and output layer and a single hidden layer. The number of neurons in the hidden layer was varied from 2 to 20 for most of the 15% scans, and from 10 to 45 for the other scans where the training sets were larger. Since the initial weights and biases can affect training, for each choice of the number of hidden neurons, 200 neural network models were constructed with random initialization of weights and biases. The predictions from each of these 200 trained models were treated as different trials of the same experiment, and the score for each mutant was calculated as the average of the 200 model predictions. *R*^2^ value for the combined set of training and validation data was monitored with the increase in the number of hidden neurons as illustrated in Fig 22B in S2 File. The number of hidden neurons was then chosen as the one with which the *R*^2^ value is the highest (Optimal number of hidden neurons given in Table 6 in S1 File). In all these above mentioned calculations we used Levenberg-Marquardt algorithm with mean square error as the performance function for training the network. Early stopping criterion was used to prevent overtraining. The parameters performance goal (*trainParam.goal*), the minimum performance gradient (*trainParam.min_grad*) and maximum number of validation fails before the training is stopped (*trainParam.max_fail*) were set to 10^−7^, 10^−8^ and 100 respectively. Default values in the *trainlm* algorithm of Neural Network Toolbox of Matlab R2017b was used for all other parameters.

### Models with reduced set of variables

The important variables were identified in two ways: (1) Assuming a linear relation between the fitness and input variable, the fraction of variance in the fitness data explained by the input variable is calculated as the square of the Pearson correlation between the input and fitness. Variables with the fraction of variance explained more than 0.1 were chosen to develop the model and were conservation, average correlation, average commute time, contacts, BLOSUM, SASA and PSSM score for the wild type amino acid; (2) Neural network models were developed by fixing each of the inputs to its average value and the percentage increase in the mean squared error upon this is used to quantify variable importance. 6 important variables were chosen based on this: impact, average correlation, average commute time, contacts, BLOSUM and hydrophobicity of the substituted amino acid.

## Supporting information

S1 File Supplementary tables. S1 File contains **Table 1**. RMSD and Pearson correlation for the test set of scans varying the number of training data points, **Table 2**. RMSD and Pearson correlation for the test set of the 15% scans, **Table 3**. Correlation of input variables with the EVmutation score, **Table 4**. Comparison of prediction quality of Deep2Full with other methods that used partial deep mutational scan data to complete the map, **Table 5**. Comparison of prediction quality of models developed in this study with that of existing methods that do not use partial data for generating the model, **Table 6**. The optimum number of hidden neurons for different scans.
(PDF)

S2 File Supplementary figures. S2 File contains **Fig 1**. Predicted mutational effect scores of β-lactamase in the colormap representation, **Fig 2**. Quality of predictions for Random 85% scan of β-lactamase at different sections of data, **Fig 3**–**Fig 5**. Predicted versus experimental fitness scores for random scans (15%, 25%, 50%, 85%) for β-lactamase, APH(3′)-II, Hsp90, MAPK1, UBE2I and TPK1, **Fig 6**. Predicted versus experimental fitness for alanine scan, **Fig 7**–**Fig 9**. Predictions of models trained on mutations chosen with different strategies at 15% data completeness for β-lactamase, APH(3′)-II, Hsp90, MAPK1, UBE2I and TPK1, **Fig 10**. Comparison of predictions of fitness at different drug concentrations with experimental fitness, **Fig 11**. Comparison of predictions of models with fewer features with that of the model with all 17 features, **Fig 12**. Predicted versus experimental fitness scores for the ANH scan of β-lactamase sorted according to the wild type amino acid, **Fig 13**. Predicted versus experimental fitness scores for the ANH scan of β-lactamase sorted according to the substituted amino acid, **Fig 14**. Distribution of prediction error along SASA, **Fig 15**. Quality of predictions versus quality of training data, **Fig 16**. Change in test set prediction quality as the percentage of data used for training is varied, **Fig 17**. RMSD of test set calculated across the range of experimental fitness for all the six proteins, **Fig 18**. Variation in the test set RMSD with the number of data points used for training calculated for different ranges of experimental fitness, **Fig 19**. Comparison of the predictions using model generated by training on randomly selected variants from the complete data with that of the model developed using randomly selected variants achievable through single nucleotide substitutions (SNS), **Fig 20**. Comparison of mutational effect score predictions for β-lactamase from unsupervised methods with experimental score, **Fig 21**. Distribution of SASA in the complete data set and the training set of random 15% scan and **Fig 22**. Demonstration of the selection of optimal number of hidden neurons.
(PDF)

S3 File Zip folder containing the Matlab code, variables used as inputs to the neural network and predictions from the neural network models built using different strategies for the six proteins discussed in the article. (ZIP)
