# Supplementary material for: Deep2Full: Evaluating strategies for selecting the minimal mutational experiments for optimal computational predictions of deep mutational scan outcomes
Source: PLoS One. 2020 Jan 10;15(1):e0227621. doi: 10.1371/journal.pone.0227621 (PMC6954071; doi:10.1371/journal.pone.0227621)
Supplement: S1 File — contains Table 1. RMSD and Pearson correlation for the test set of scans varying the number of training data points, Table 2. RMSD and Pearson correlation for the test set of the 15% scans, Table 3. Correlation of input variables with the EVmutation score, Table 4. Comparison of prediction quality of Deep2Full with other methods that used partial deep mutational scan data to complete the map, Table 5. Comparison of prediction quality of models developed in this study with that of existing methods that do not use partial data for generating the model, Table 6. The optimum number of hidden neurons for different scans. (PDF) [file pone.0227621.s001.pdf]

Supplementary Information

Deep2Full: Evaluating strategies for selecting the minimal mutational experiments for optimal computational predictions of deep mutational scan outcomes

C. K. Sruthi[1] and Meher K. Prakash[1]*

Theoretical Sciences Unit, Jawaharlal Nehru Centre for Advanced Scientific Research

Bangalore-560064, India

Tables

	RMSD					Pearson correlation				
Scan / Protein	Random 15%	Random 25%	Random 50%	Random 85%	SNS-Random 25%	Random 15%	Random 25%	Random 50%	Random 85%	SNS-Random 25%
β-lactamase	0.67	0.64	0.57	0.54	0.65	0.81	0.83	0.87	0.89	0.83
APH(3')-II	1.11	1.12	1.05	0.98	1.18	0.69	0.68	0.72	0.78	0.68
Hsp90	0.22	0.20	0.19	0.17	0.21	0.72	0.77	0.82	0.85	0.75
MAPK1	0.41	0.40	0.35	0.33	0.42	0.62	0.63	0.74	0.77	0.63
UBE2I	0.33	0.30	0.28	0.27	0.30	0.52	0.59	0.66	0.67	0.61
TPK1	0.39	0.39	0.38	0.35	0.42	0.24	0.23	0.26	0.42	0.24

Table 1. RMSD and Pearson correlation for the test set of scans varying the number of training data points.

	RMSD					Pearson correlation				
Scan / Protein	ANH Scan	Random 15%	Position range scan	WT residue type scan	SASA range scan	ANH Scan	Random 15%	Position range scan	WT residue type scan	SASA range scan
β-lactamase	0.71	0.67	0.92	0.90	1.03	0.80	0.81	0.65	0.67	0.53
APH(3')-II	1.13	1.11	1.32	1.31	1.39	0.67	0.69	0.52	0.54	0.49
Hsp90	0.22	0.22	0.39	0.31	0.33	0.75	0.72	0.30	0.37	0.50
MAPK1	0.42	0.41	0.57	0.52	0.55	0.62	0.62	0.31	0.39	0.33
UBE2I	0.31	0.33	0.46	0.40	0.41	0.56	0.52	0.20	0.32	0.31
TPK1	0.39	0.39	0.45	0.40	0.43	0.25	0.24	0.13	0.19	0.10

Table 2. RMSD and Pearson correlation for the test set of the 15% scans.

Variable	Pearson correlation with EVmutation
Conservation	-0.49
SASA	0.39
Contacts	-0.36
Average commutetime	0.34
Average co-evolution	-0.32
Closeness centrality	-0.27
Eigenvector centrality	-0.25
Degree centrality	-0.23

Table 3. Table showing the Pearson correlation of different variables that was considered in our study as inputs for the neural network with the EVmutation score. Negative values indicate anti-correlation.

	Pearson correlation		RMSD	
	Random 85%	Envision	Random 85%	Roth et al.
β-lactamase	0.89	0.85	0.54	-
APH(3')-II	0.78	0.84	0.98	-
Hsp90	0.85	0.76	0.17	-
UBE2I	0.67	-	0.27	0.24
TPK1	0.42	-	0.35	0.34

Table 4. Comparison of prediction quality of Deep2Full with other methods which used partial deep scan data to complete the map. For Envision the Pearson correlation for the test set of individual protein models developed by training on 80% of deep mutational scan data was obtained from Figure 2 of *Gray et al.*[1].

	Spearman correlation								
Protein	Deep-Sequence	EVmutation	SNAP2	Envision	ANH scan	Random 15%	Random 25%	Random 50%	Random 85%
β-lactamase	0.78	0.72	0.71	0.74*	0.80	0.81	0.83	0.86	0.88
APH(3')-II	0.59	0.54	0.49	0.64*	0.67	0.68	0.67	0.72	0.77
Hsp90	0.53	0.49	0.43	0.31*	0.53	0.56	0.59	0.65	0.70
MAPK1	-0.24	-0.25	0.30	-0.44	0.60	0.59	0.60	0.71	0.75
UBE2I	0.55	0.51	-0.51	0.09	0.56	0.52	0.59	0.65	0.66
TPK1	0.26	0.25	-0.22	0.27	0.25	0.24	0.24	0.26	0.42

Table 5. Comparison of prediction quality of our models with that of existing methods which do not use partial data for generating the model. For DeepSequence[2] and EVmutation[3], the data was taken from the supplementary information of *Riesselman et al.*[2]. *Extracted from the supplementary figure 8 on Leave-One-Protein-Out analysis of *Gray et al.*[1].

Scan / Protein	Random 85%	Random 50%	Random 25%	Random 15%	ANH scan	Position range scan	WT residue type scan	SASA range scan
blact	41	31	30	15	13	20	20	20
agk	26	33	28	15	15	20	12	20
hsp90	42	30	12	12	11	20	9	20
mapk1	35	21	28	19	20	17	15	20
ube2i	16	12	40	25	23	20	14	18
tpk1	40	40	36	15	20	20	15	14

Table 6. Optimal number of hidden neurons for all proteins and scans

References

[1] Gray VE, Hause RJ, Luebeck J, Shendure J, Fowler DM. Quantitative missense variant effect prediction using large-scale mutagenesis data. Cell systems. 2018;6(1):116–124.

[2] Riesselman AJ, Ingraham JB, Marks DS. Deep generative models of genetic variation capture the effects of mutations. Nature Methods. 2018;15(10):816+. doi:10.1038/s41592-018-0138-4.

[3] Hopf TA, Ingraham JB, Poelwijk FJ, Scharfe CPI, Springer M, Sander C, et al. Mutation effects predicted from sequence co-variation. Nature Biotechnology. 2017;35(2):128–135. doi:10.1038/nbt.3769.
