# Supplementary material for: Deep2Full: Evaluating strategies for selecting the minimal mutational experiments for optimal computational predictions of deep mutational scan outcomes
Source: PLoS One. 2020 Jan 10;15(1):e0227621. doi: 10.1371/journal.pone.0227621 (PMC6954071; doi:10.1371/journal.pone.0227621)
Supplement: S2 File — contains Fig 1. Predicted mutational effect scores of β-lactamase in the colormap representation, Fig 2. Quality of predictions for Random 85% scan of β-lactamase at different sections of data, Fig 3–Fig 5. Predicted versus experimental fitness scores for random scans (15%, 25%, 50%, 85%) for β-lactamase, APH(3′)-II, Hsp90, MAPK1, UBE2I and TPK1, Fig 6. Predicted versus experimental fitness for alanine scan, Fig 7–Fig 9. Predictions of models trained on mutations chosen with different strategies at 15% data completeness for β-lactamase, APH(3′)-II, Hsp90, MAPK1, UBE2I and TPK1, Fig 10. Comparison of predictions of fitness at different drug concentrations with experimental fitness, Fig 11. Comparison of predictions of models with fewer features with that of the model with all 17 features, Fig 12. Predicted versus experimental fitness scores for the ANH scan of β-lactamase sorted according to the wild type amino acid, Fig 13. Predicted versus experimental fitness scores for the ANH scan of β-lactamase sorted according to the substituted amino acid, Fig 14. Distribution of prediction error along SASA, Fig 15. Quality of predictions versus quality of training data, Fig 16. Change in test set prediction quality as the percentage of data used for training is varied, Fig 17. RMSD of test set calculated across the range of experimental fitness for all the six proteins, Fig 18. Variation in the test set RMSD with the number of data points used for training calculated for different ranges of experimental fitness, Fig 19. Comparison of the predictions using model generated by training on randomly selected variants from the complete data with that of the model developed using randomly selected variants achievable through single nucleotide substitutions (SNS), Fig 20. Comparison of mutational effect score predictions for β-lactamase from unsupervised methods with experimental score, Fig 21. Distribution of SASA in the complete data set and the training set of random 15% [file pone.0227621.s002.pdf]

Supplementary Information

Deep2Full: Evaluating strategies for selecting the minimal mutational experiments for optimal computational predictions of deep mutational scan outcomes

C. K. Sruthi[1] and Meher K. Prakash[1]*

[1] *Theoretical Sciences Unit*

Jawaharlal Nehru Centre for Advanced Scientific Research

Bangalore - 560064, India

Figures

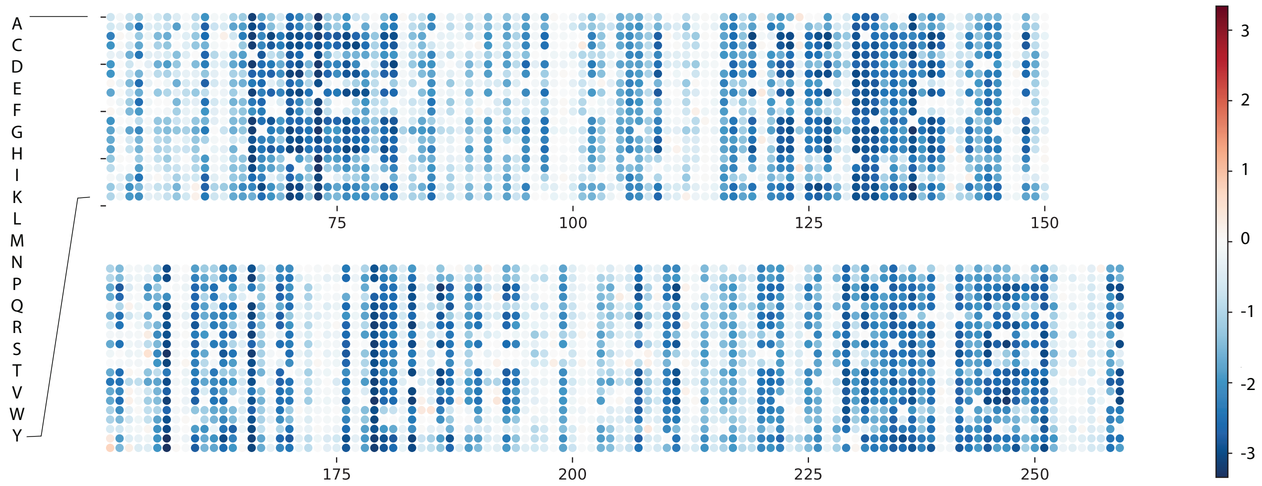

Fig 1. Computational fitness map: Computational predictions of the quantitative gain or loss in fitness of *E. coli* resulting from amino acid changes in β-lactamase when challenged with 2500 μg/ml concentration of ampicillin. The panel includes the training, validation and test sets. In this specific case the training and validation sets add up to 85% of the mutational scan data.

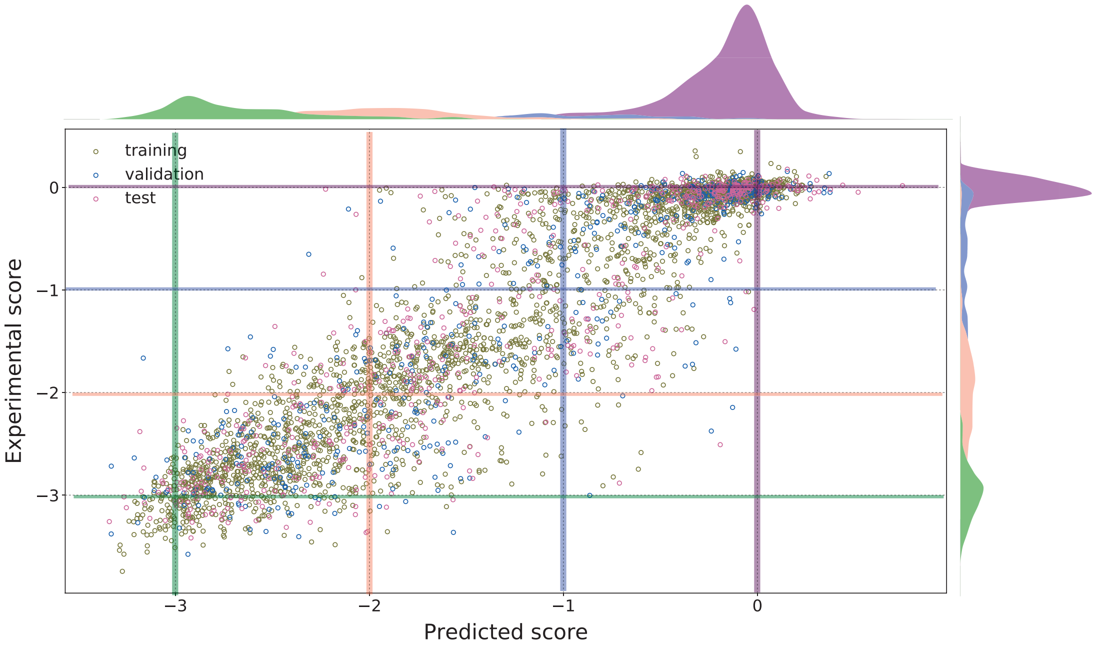

Fig 2. Distribution of predictions over the range of fitness: Quality of predictions when the model was trained with 85% data is shown at different sections of the data. Distributions were constructed by taking data which are 0.3 units wide (0.15 to -0.15, -1.15 to -0.85, -2.15 to -1.85 and -3.15 to -2.85). The predictions made around fitness score 0 or predicted as neutral are more reliable as the spread in the experimental data corresponding to these predictions is lower.

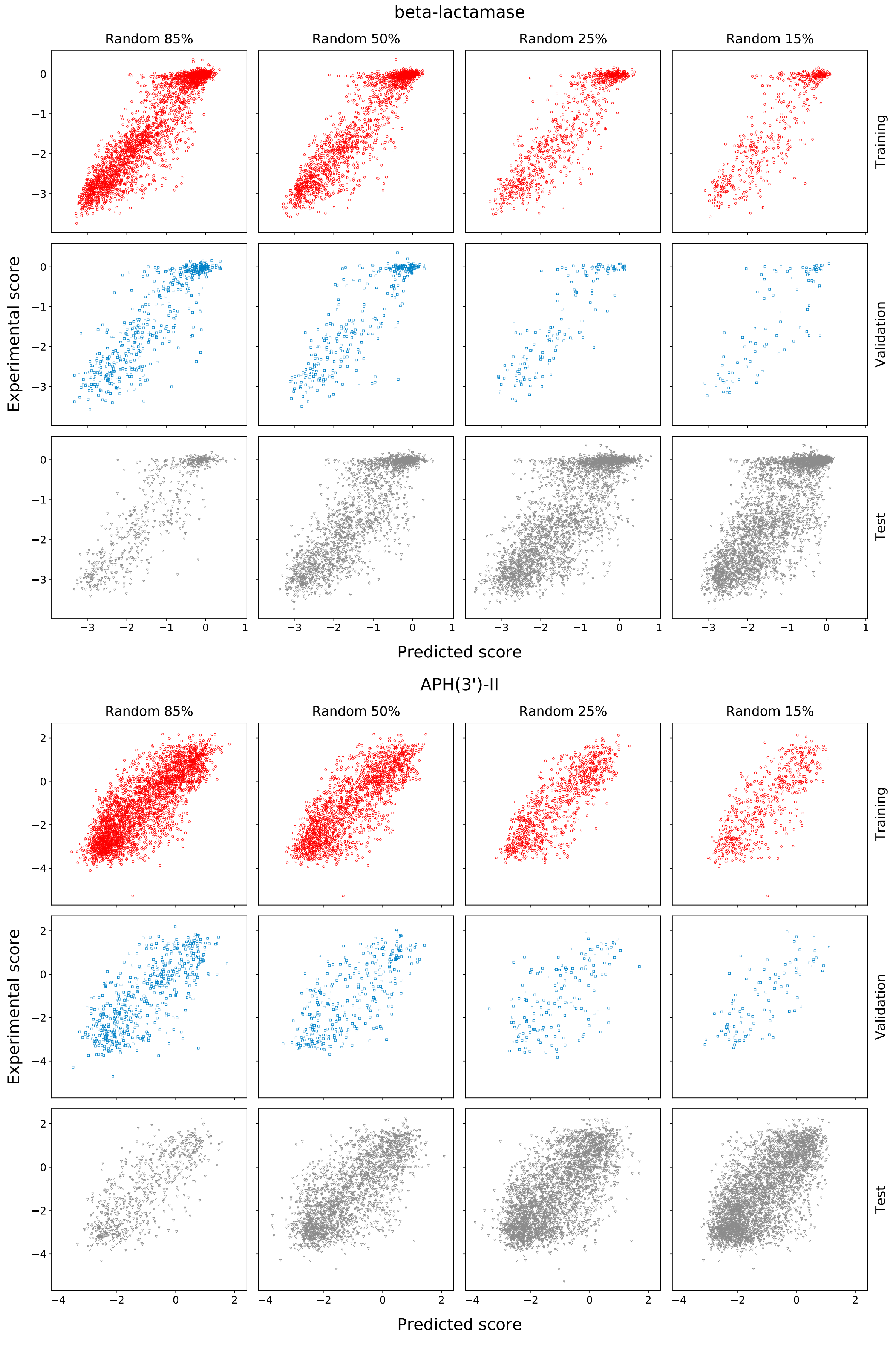

Fig 3. Results from training the model with systematically reduced data sets: Fitness scores obtained computationally by training the models on decreasing sizes of data sets. Training, validation and test sets for β-lactamase and APH(3′)-II are shown. The RMSD and Pearson correlation for the test set for all proteins and for all scans are given in Table 1 in S1 File.

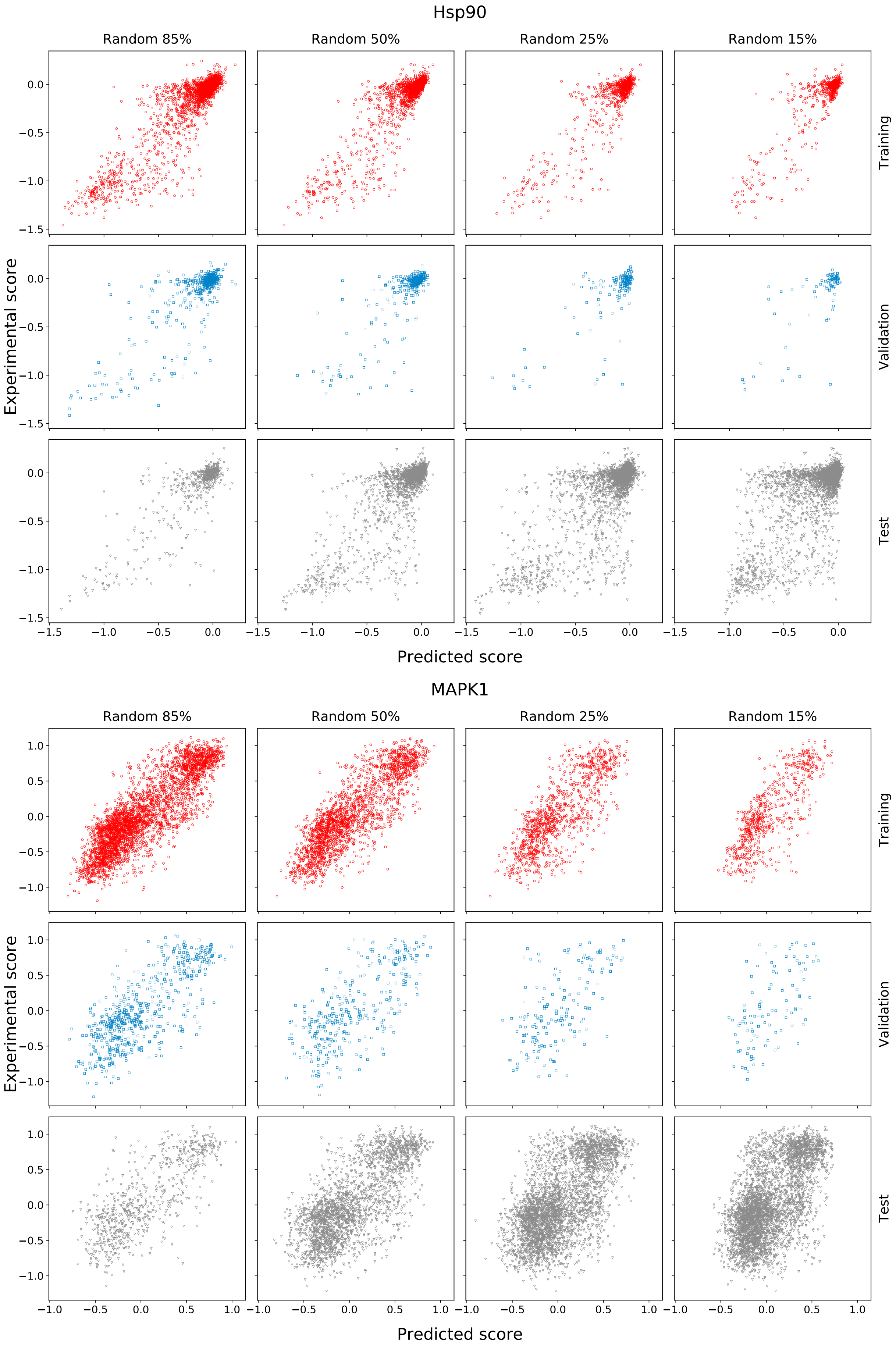

Fig 4. Hsp90 and MAPK1

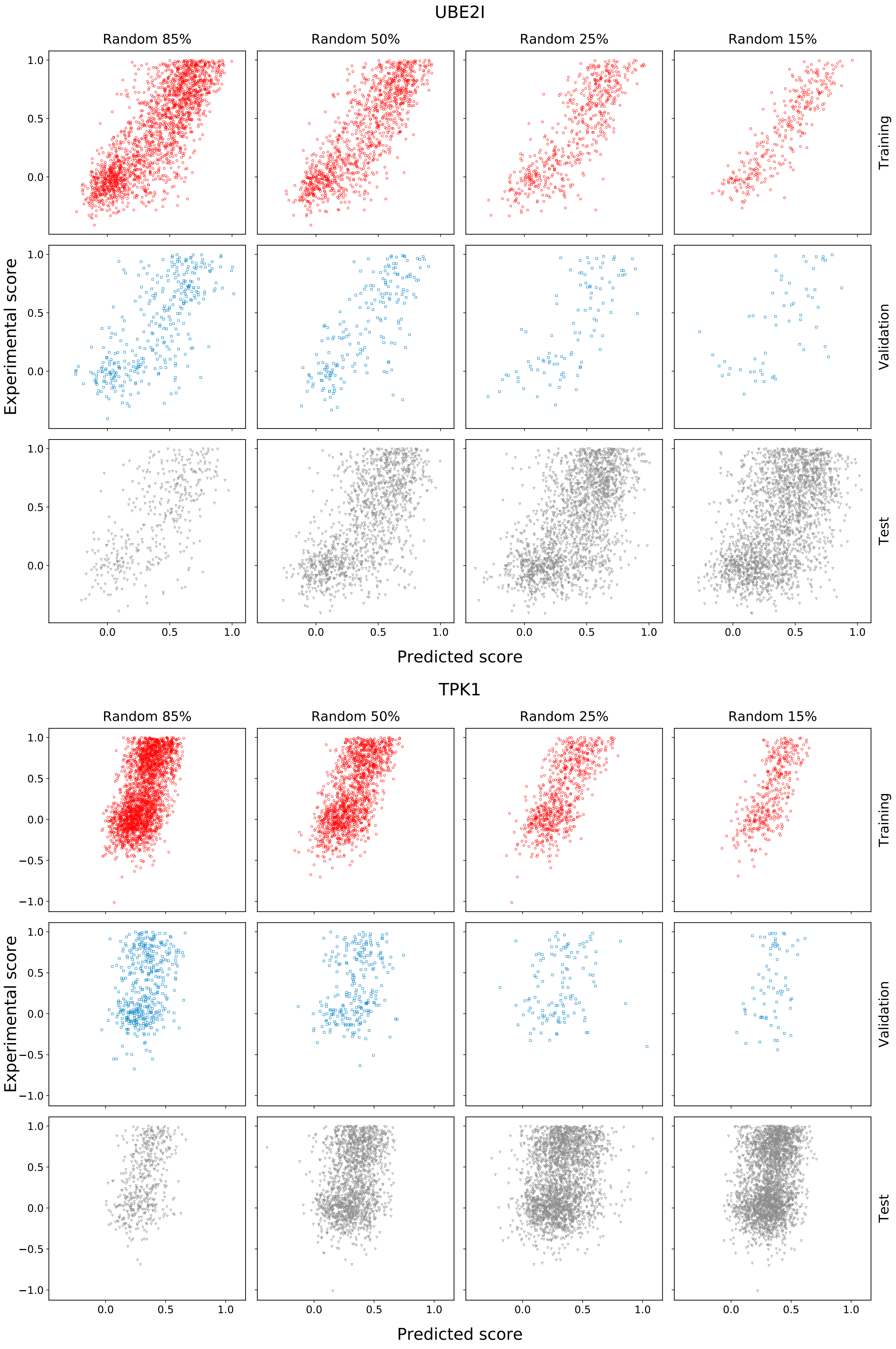

Fig 5. UBE2I and TPK1

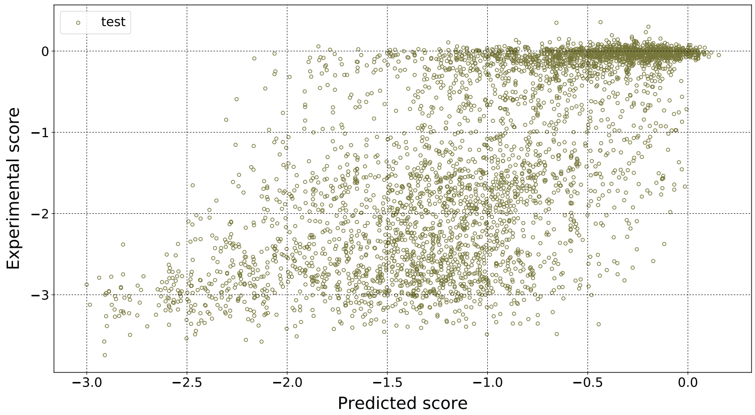

Fig 6. Insufficiency of alanine scan for training the model: The experimental fitness data on substitution at every amino acid position of β-lactamase with alanine was used for training the model. This strategy which only used 5% of the full mutational data for predicting the fitness of all other 19 mutational scans did not give good predictions. We did not use alanine scan for any further analysis.

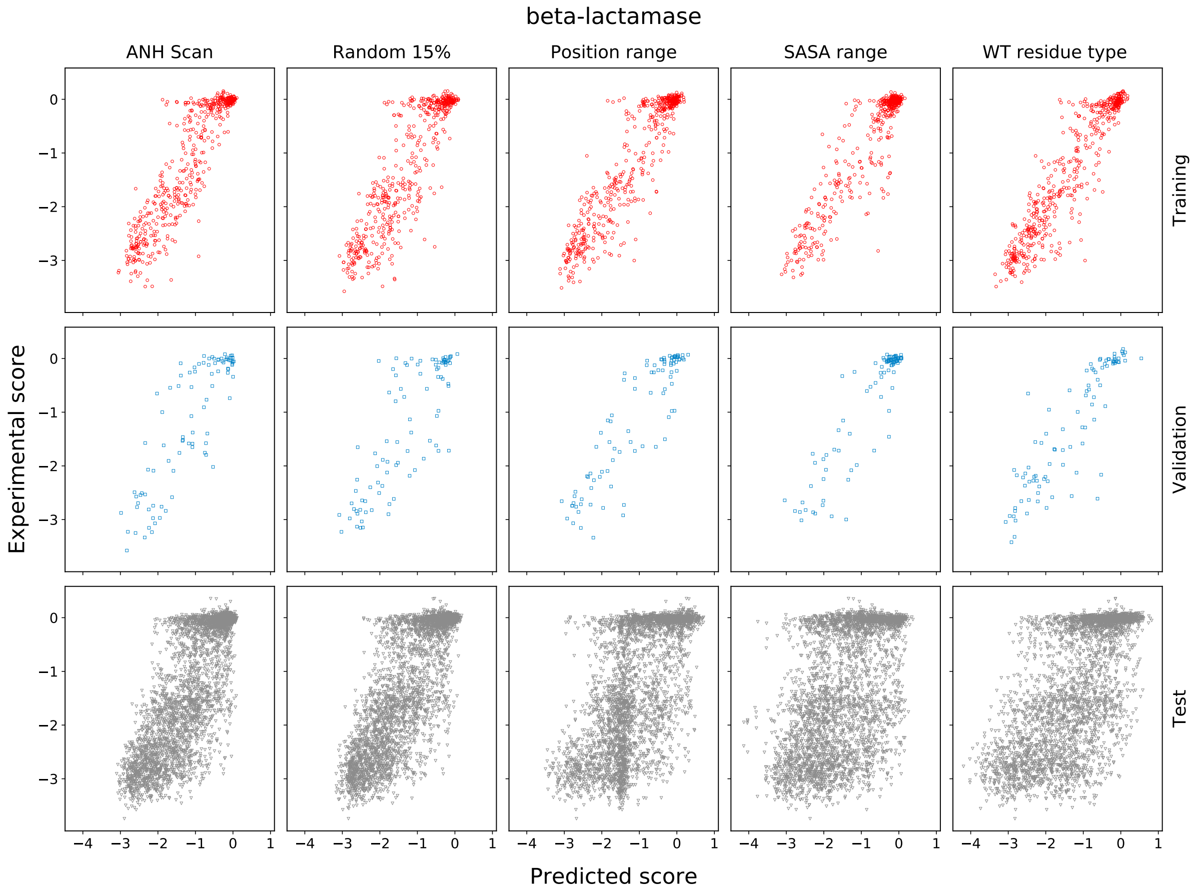

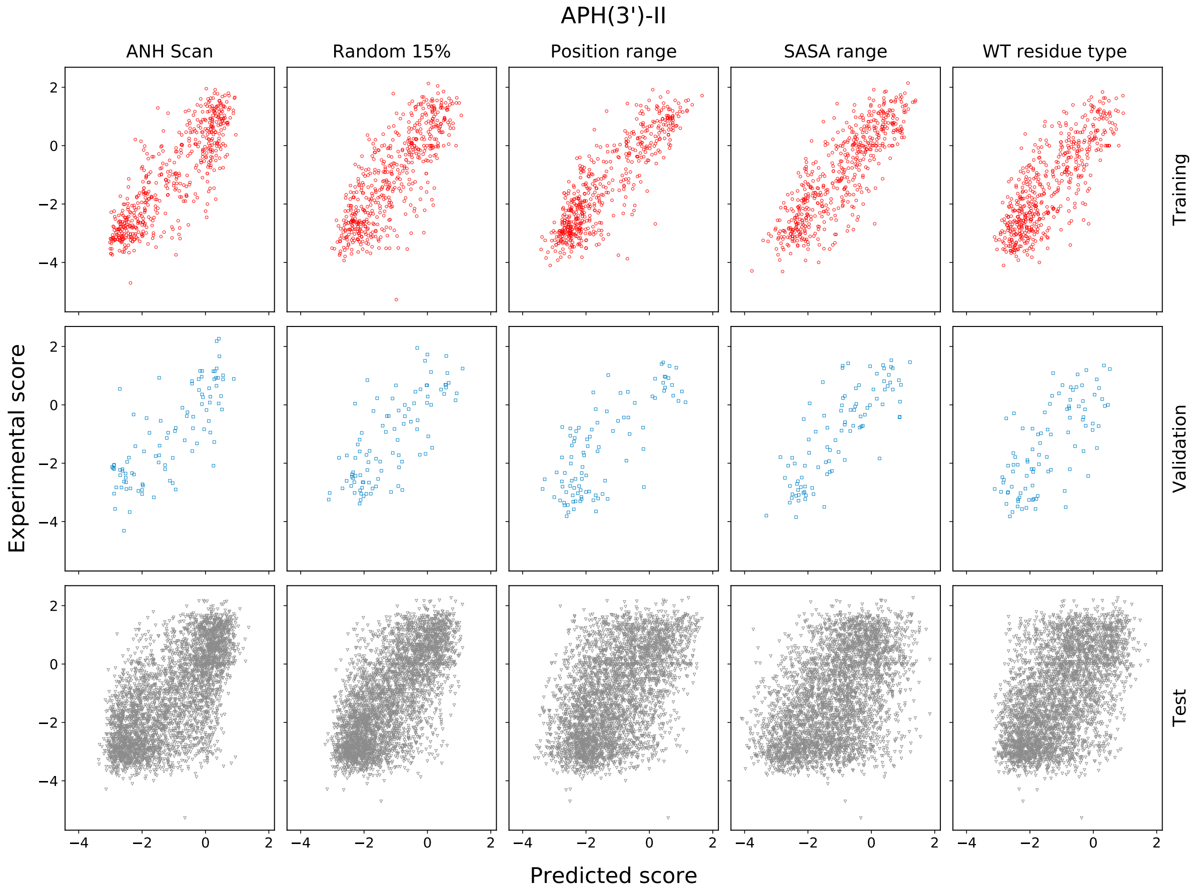

Fig 7. Results from training the model on mutations chosen with different strategies: Fitness scores obtained computationally by training the models on 15% of data chosen in different ways. The training, validation and test sets for β-lactamase and APH(3′)-II are shown. The RMSD and Pearson correlation for the test set for all proteins and for all scans are given in Table 1 in S1 File.

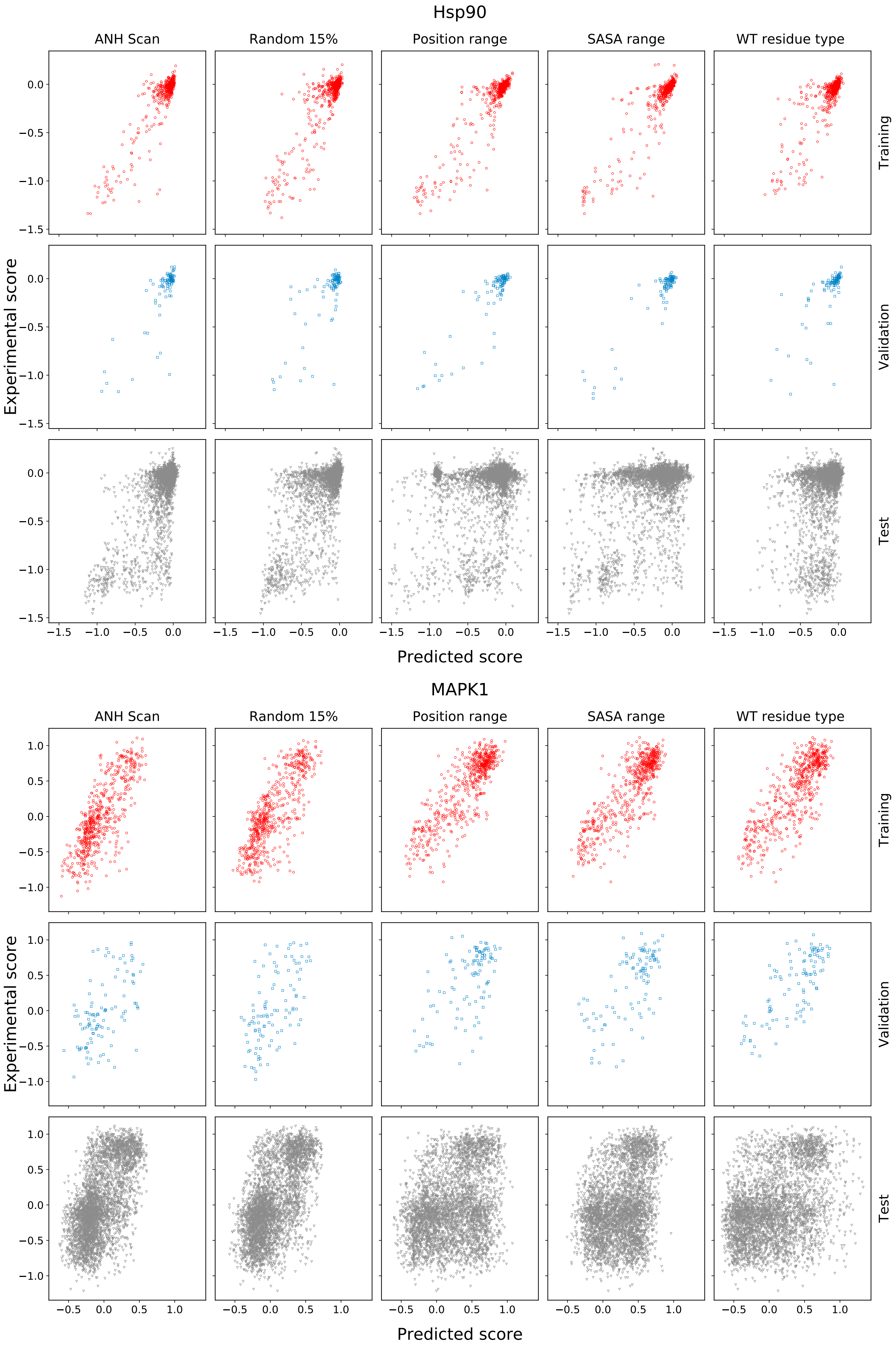

Fig 8. Hsp90 and MAPK1

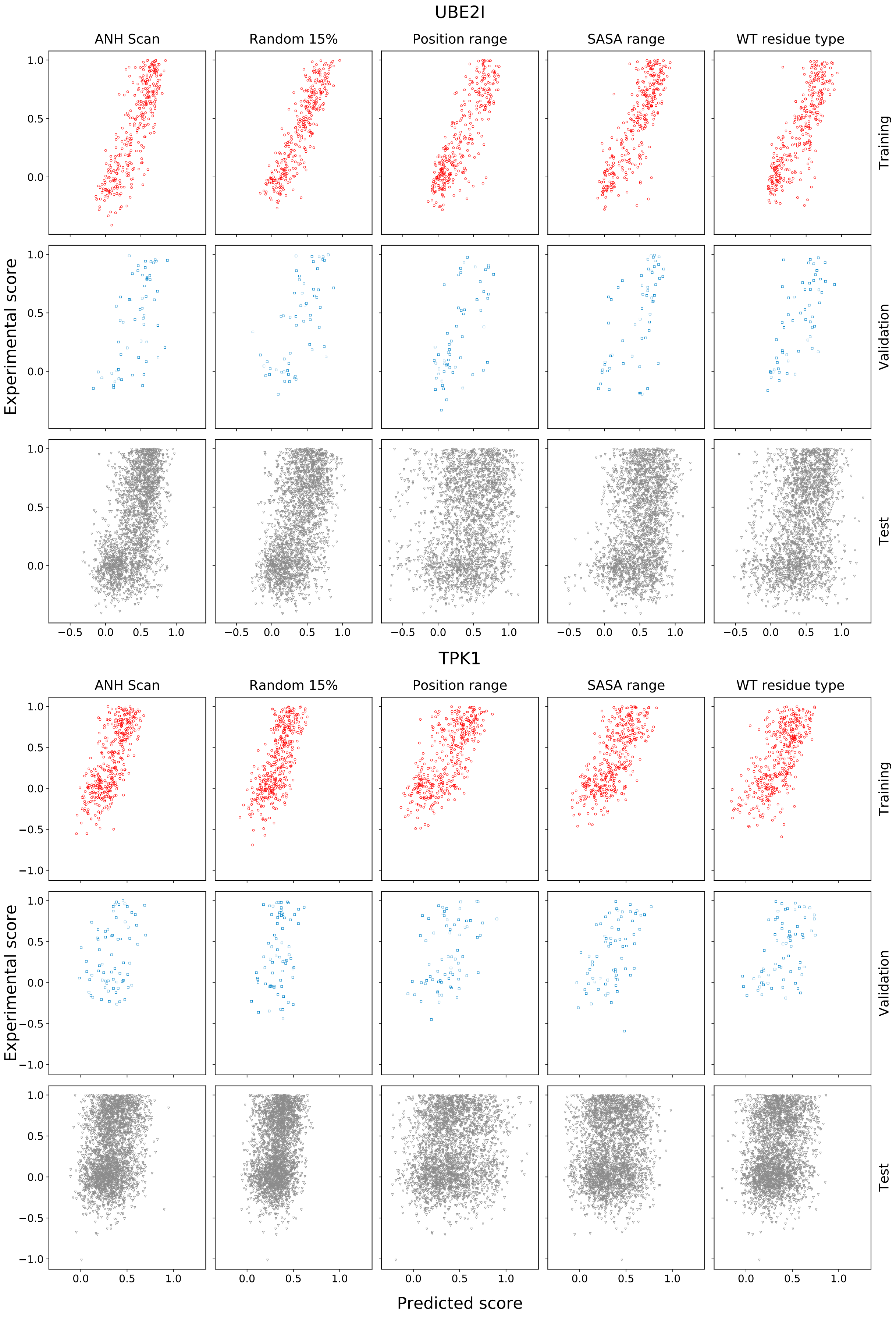

Fig 9. UBE2I and TPK1

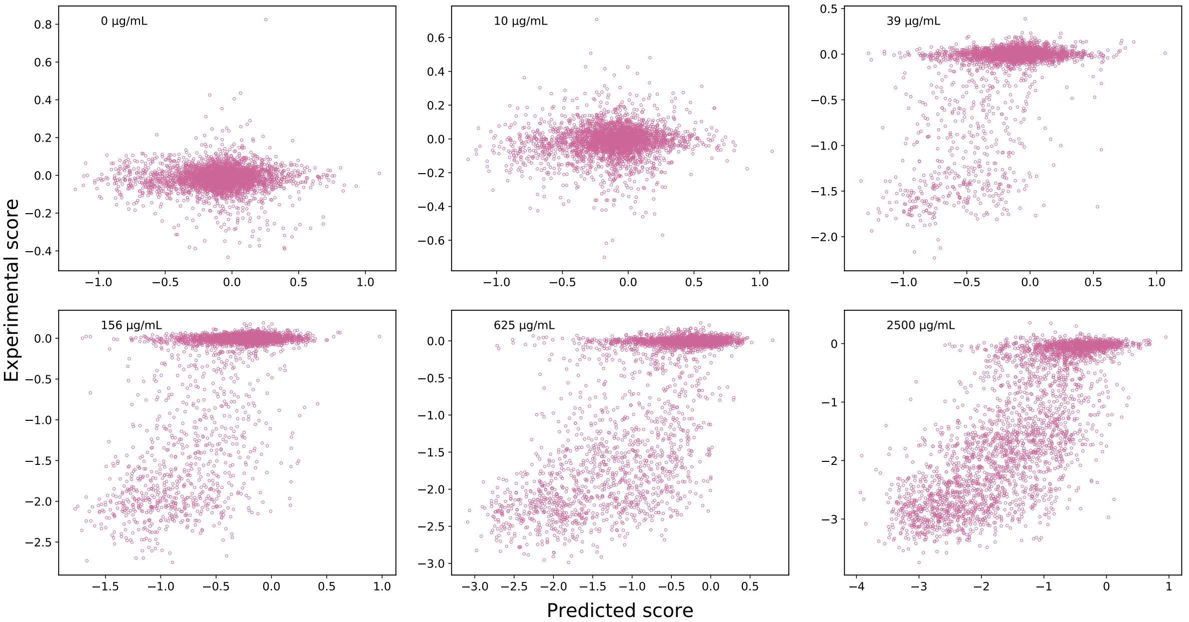

Fig 10. Results from augmenting the data with transverse assay conditions: For the same amino acid substitutions we included the fitness data obtained at different antibiotic concentrations: 0, 10, 39, 156, 625 and 2500 μg/mL in the training set. Predictions for the remaining 85% of the mutations that were not used in the training are shown and augmenting do not improve the results.

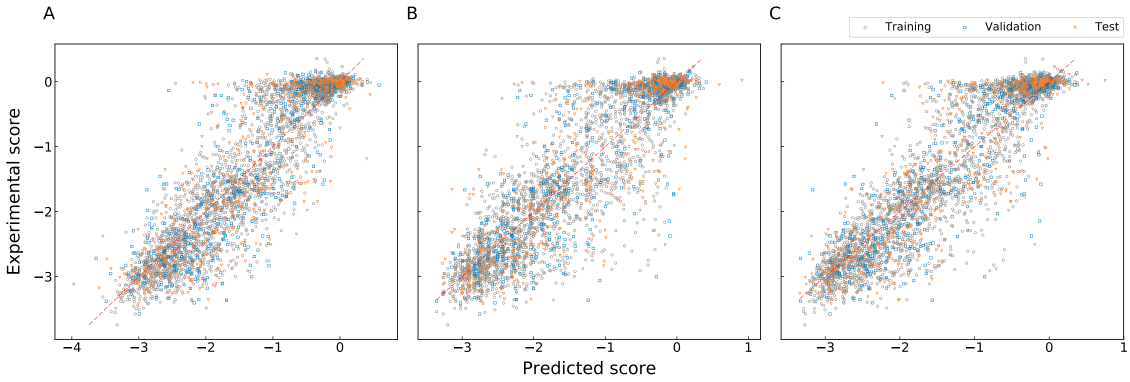

Fig 11. Feature selection: Predictions from models with reduced number of variables. (A) Using the variables impact, average correlation, average commute time, contacts, BLOSUM and hydrophobicity of mutant which were chosen based on the variable impact analysis (B) Using variables chosen based on the pearson correlation between the input variables and fitness: conservation, average correlation, average commute time, contacts, BLOSUM, SASA, PSSM score for wild type amino acid (C) Using all 17 variables. The models with fewer variables have comparable predictive abilities as that the one using all variables. The adjusted R^2 values for the test set are 0.74, and 0.74 and 0.78 respectively for A, B and C. The variables average correlation, average commute time, contacts and Blosum are common in a and b.

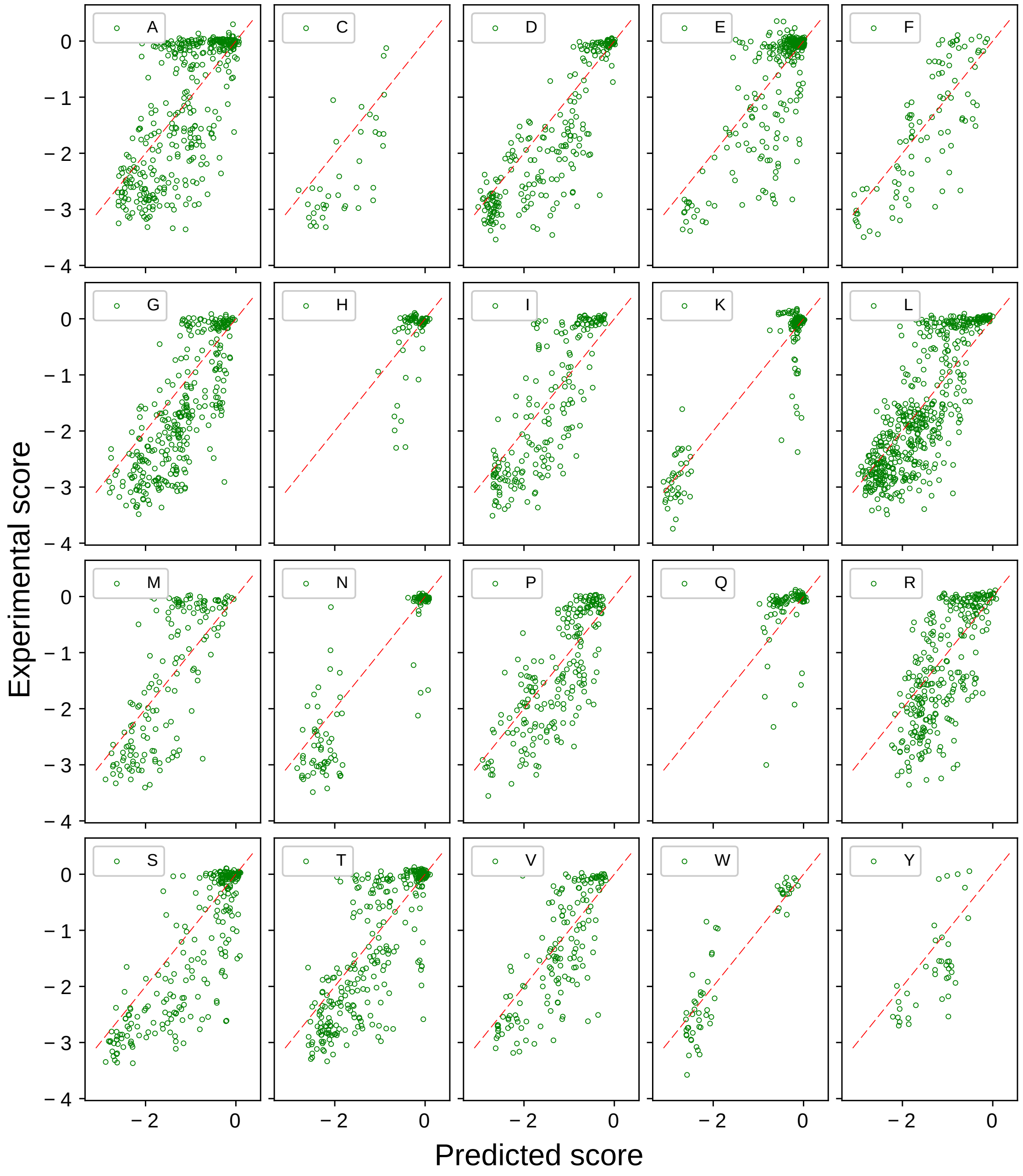

Fig 12. Prediction quality relative to the wild type amino acid: Predictions from ANH were also analyzed by classifying them according to the wild type amino acid *which gets mutated*. The analysis shows that there is variability in the predictability of substitution of different amino acids such as asparagine (N) and tryptophan (W) having high prediction quality and histidine (H) and glutamine (Q) relatively poor. The dashed red lines are guidelines with slope 1.

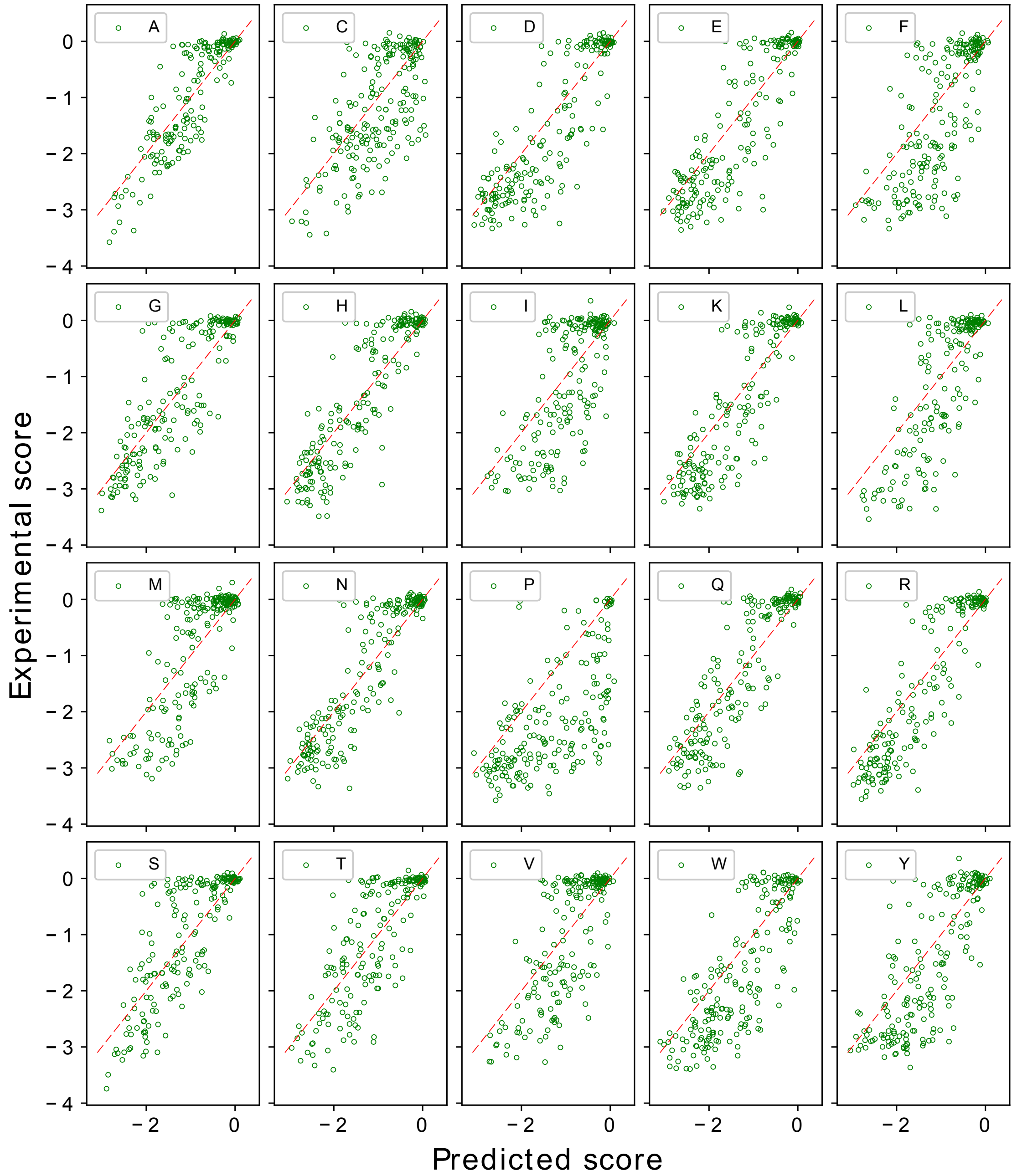

Fig 13. Quality of output relative to the substituted amino acid: Predictions from ANH were analysed by classifying them according to the amino acid to which *mutation is performed*. It can be seen that the predictability of all amino acids is comparable. However, the predictions to alanine (A), asparagine (N), histidine (N) are notably better because of the training set that was used. The dashed red lines are guidelines with slope 1.

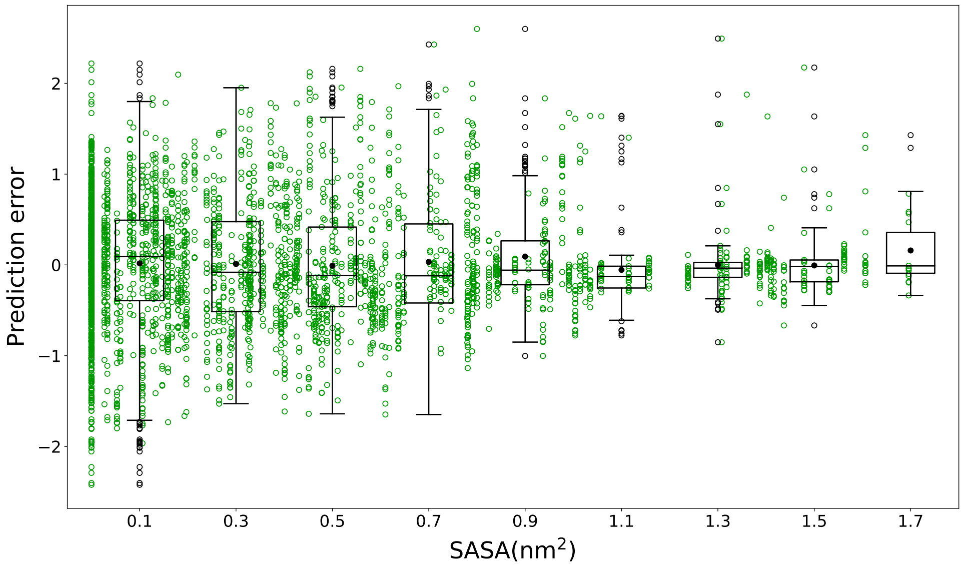

Fig 14. Distribution of error along SASA: Prediction error (Predicted fitness - experimental fitness) for the test set of random 15% scan for β-lactamase as a function of solvent exposure. The box plot shows the distribution of errors at different values of SASA. At higher values of SASA, error is lower.

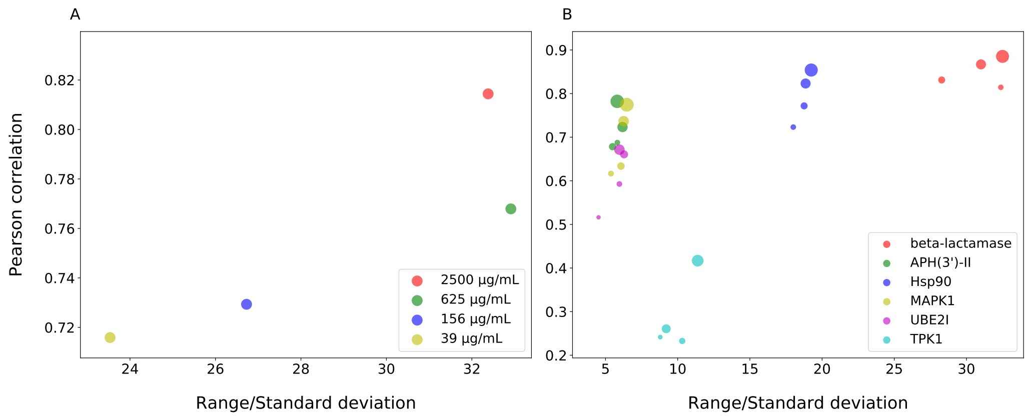

Fig 15. Output quality versus input quality: Quality of data used in training defined as the ratio of the range of mutational effect scores in the training set and the standard deviation of the mode corresponding to the neutral substitutions with the prediction quality shown in Pearson correlation between the predictions and measured fitness for the test set was used as a measure of the quality of output. Quality of input vs and quality of output is compared for (A) models developed for mutational effect scores measured for β-lactamase under different concentrations of ampicillin (B) random scans performed for the six proteins. Comparison in the same system has a trend, while no clean trend could be seen in the comparisons across different protein and scans.

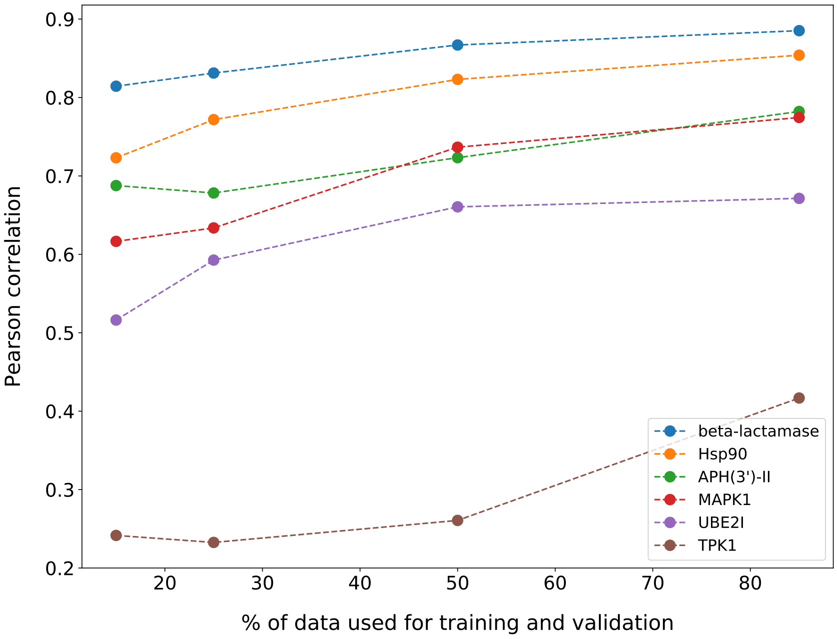

Fig 16. Quality of predictions using different random scans: The quality of predictions as seen from the Pearson correlation between predicted and experimental data in general improve for all proteins. This improvement slows down beyond 50% data usage except for TPK1.

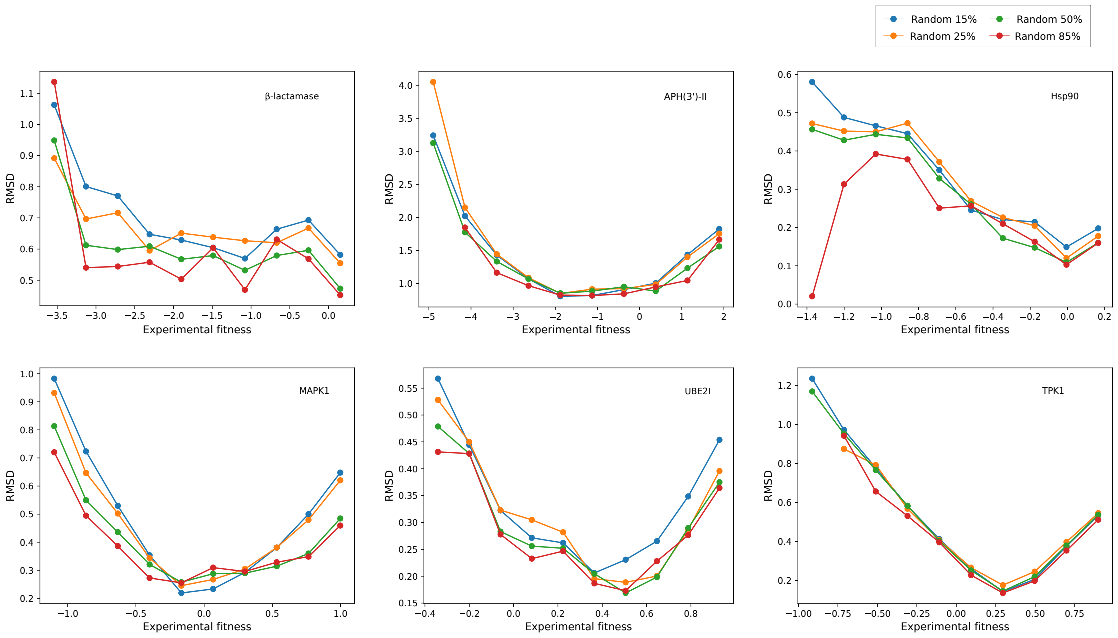

Fig 17. Distribution of error over the range of fitness: RMSD of test set calculated across the range of experimental fitness for all the six proteins. The experimentally observed fitness was divided into 10 bins in each of the cases and RMSD was quantified for each bin. As it can be seen, errors are fairly systematic, which suggests that the RMSD relative to the expected values is fairly a constant for several of the cases.

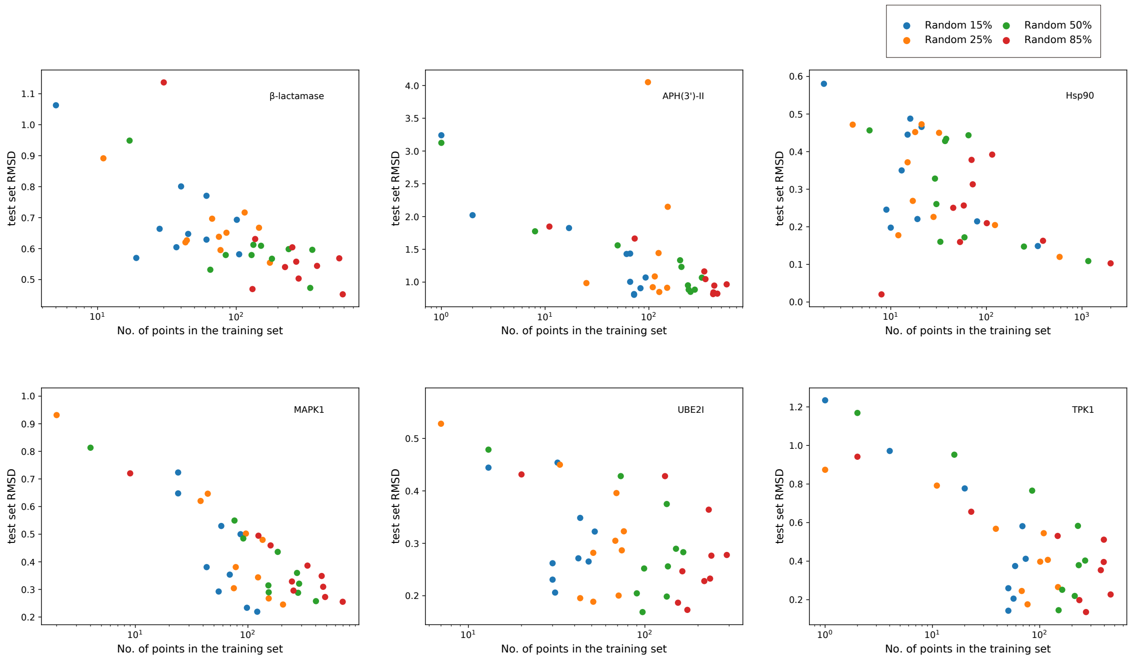

Fig 18. Reduction of error with the increasing number of training points per bin: RMSD of test set calculated for different ranges of the experimental fitness scores versus the number of points in the training set in each bin. A power-law behavior emerges. It can also be seen that having more than 100 points for training from a bin does not improve the predictions significantly, which suggests an approximate 100×10 bins = 1000 data points for training.

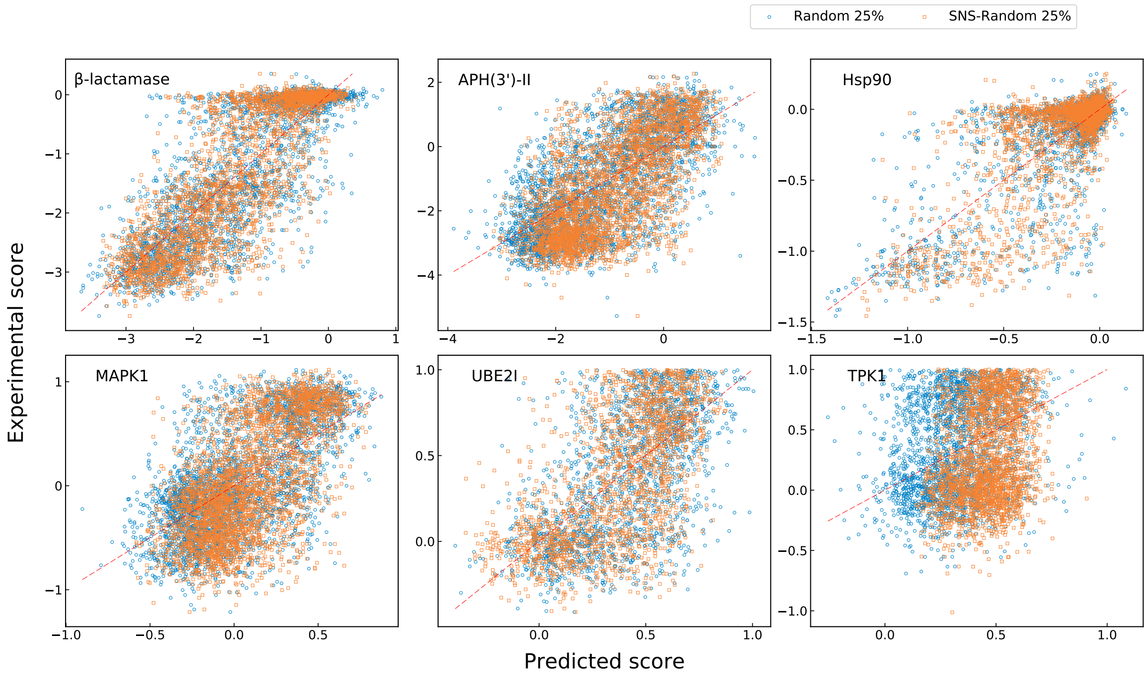

Fig 19. Comparing the results of randomly choosing from SNS versus randomly choosing from all possible substitutions: Comparison of quality of predictions by models generated by training on randomly selected variants from the complete data and randomly selected mutations achievable through single nucleotide substitutions (SNS). 25% of data was used for training and validation. The RMSD and Pearson correlation for the test set for both scans and for all proteins are given in Table 1 in S1 File. It can be seen that the performance of both models are comparable.

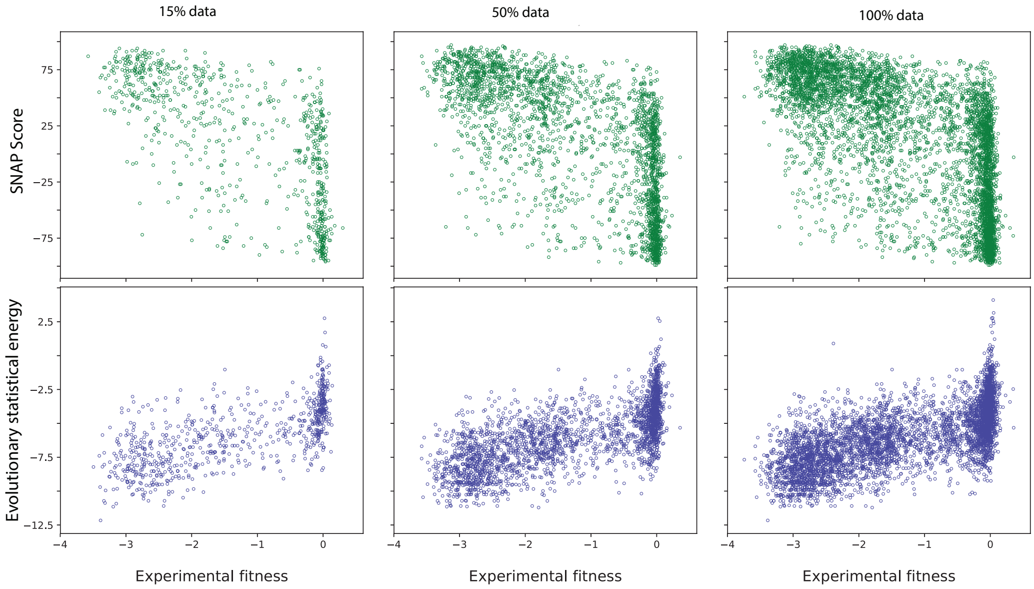

Fig 20. Comparison of mutational effect score predictions from unsupervised methods with the experimental score: A comparison of how the scores from SNAP2 and Evolutionary statistical energy relate to the experimentally observed fitness of *E. coli* arising from β-lactamase mutations is shown in this figure. The scores are compared for the complete experimental data as well as by randomly selecting 15%, 50% of the data.

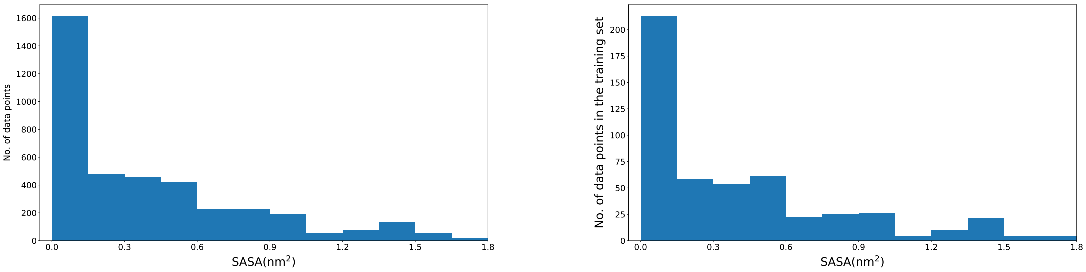

Fig 21. Random scan is representative: Distribution of SASA for the complete data as well as the training set of random 15% scan for β-lactamase. The distributions are similar showing that the random choice of mutations is representative of the complete data set.

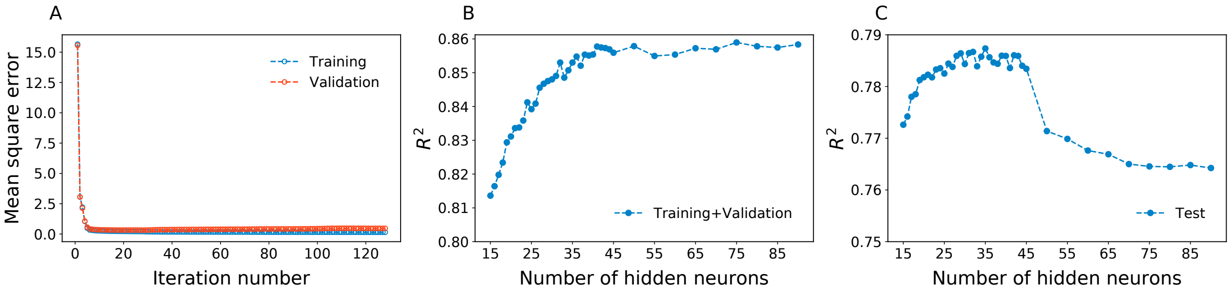

Fig 22. Convergence of neural network model training and test: Results demonstrating how the choice of number of hidden neurons was made for β-lactamase for the random 85% scan. **(A)** For a given choice for the number of neurons in the hidden layer, 41 in this case, mean square error calculated for the training set as well as the validation set as the iteration proceeds. The quality of predictions for training data improves, and beyond the optimal number of iterations, the error of predictions for the validation set increases. To avoid this problem of overtraining early stopping criterion was used to terminate the iterations. When this worsening consistently occurs for 100 iterations, the training is terminated. The iteration at which the validation set has the lowest error is then chosen. **(B)** Similar calculation was repeated by changing the number of neurons, and the R^2 for training and validation was monitored as a function of the number of neurons. The decision about the optimal number of neurons was made considering the performance both in the training and the validation sets. In this case, 41 neurons in the hidden layer was considered optimal. **(C)** R^2 value for test set, obtained by performing the calculations at any given number of neurons is also shown. In this specific case, this graph is used an *a posteriori* justification for our choice of 41 neurons in the hidden layer. As it can be seen although with higher number of neurons the training and validation may saturate, the test set can worsen, and attention needs to be paid to avoid this problem by over training.
